# Growth responses of spring barley to varying levels of drought priming

**DOI:** 10.3389/fpls.2025.1716430

**Published:** 2026-01-22

**Authors:** Zohreh Salehi Soghadi, Peiman Zandi, Yaosheng Wang, Hans-Peter Kaul

**Affiliations:** 1 Department of Agricultural Sciences, Institute of Agronomy, BOKU University, Tulln, Austria; 2 State Key Laboratory of Efficient Utilization of Agricultural Water Resources, Key Laboratory of Dryland Agriculture, Ministry of Agriculture and Rural Affairs of China, Institute of Environment and Sustainable Development in Agriculture, Chinese Academy of Agricultural Sciences, Beijing, China; 3 Department of Agroecology, Aarhus University, Tjele, Denmark

**Keywords:** water deficit, *Hordeum vulgare* L., recovery dynamics, re-watering, vegetative development, water use efficiency

## Abstract

**Introduction:**

Drought-associated environmental changes pose a significant threat to global agricultural sustainability. While barley’s response to single drought events is well-documented, its adaptations to recurrent drought-rehydration cycles during the vegetative stages remain unexplored.

**Methods:**

Barely plants were subjected to five glasshouse watering regimes and harvested at six sampling times (S1-S6): full irrigation (FI, 85% soil water-holding capacity, SWHC), and four drought treatments varying in severity (mild 65% vs. severe 45% SWHC) and pattern (intermittent ID1/ID2 with rehydration vs. persistent PD1/PD2 without).

**Results:**

Persistent drought (PD1, PD2) consistently reduced interval-specific water-use efficiency (WUEn) across all stages, while intermittent drought (ID2) enhanced WUEn during tillering. Stomatal conductance (*g_s_*) was lowest in PD treatments, with PD1 exhibiting minimum values at seedling (S1) and jointing (S5), indicating severe transpiration limitation. ID2 maintained higher *g_s_* than ID1 during seedling and tillering stages (S2, S3). However, cumulative severe stress ultimately impaired stomatal regulation in ID2, leading to ID1’s superiority by the jointing stage (S5–S6). Multivariate analysis identified stress severity as the primary initial driver of physiological disruption, revealing a fundamental shift to a biochemical stress-acclimation strategy in later stages.

**Discussion:**

The findings delineate a dynamic recovery response under intermittent drought from a conservative, high-cost strategy under persistent drought. They suggest that applying more severe cyclic drought early (ID2, S1-S4) followed by milder cyclic stress later (ID1, S5-S6) optimizes stomatal function and productivity, enhancing resource-use efficiency under water limitation.

## 1 Introduction

Climate change has emerged as a pressing concern at local, regional, and global scales, posing severe threats to land areas in the form of drought and other adverse weather events (Qiu et al., 2012; Li and Geng, 2013; Aliyeva et al., 2020). Addressing such challenges is crucial to achieving Sustainable Development Goal 2, Zero Hunger, as outlined in the 2030 Agenda for Sustainable Development. This goal highlights the need to resolve the water crisis in agriculture to ensure global food security (Food and Agriculture Organization of the United Nations (FAO), 2020). Therefore, achieving high-yields under water-limited conditions, especially in dryland agricultural systems, is imperative.

Barley (*Hordeum vulgare* L.), an economically important cereal crop, is particularly relevant in this context due to its ability to adapt to drought-prone environments. Both wild and cultivated barley genotypes have shown varying capacities to withstand drought stress (Barati et al., 2015, 2020). Although barley can endure short-term droughts once established, prolonged water deficits severely impact its growth, grain yield, and quality (Paul et al., 2023). This vulnerability is especially evident in (semi)arid regions of Southern Europe, such as southern Spain and Italy (Latorre et al., 2001; Pellicone et al., 2019). Notably, barley cultivars that exhibit high yield and quality potential under favorable conditions frequently lack tolerance to water deficits in these regions (Pennisi, 2008; Elakhdar et al., 2022).

Drought stress affects barley’s physiological and morphological traits by raising its root-to-shoot ratio, promoting lateral root formation, and reducing root vessel efficiency (Li et al., 2020). Consequently, water and nutrient uptake is compromised, resulting in lower yields and grain quality. Similarly, other crops like wheat experience reduced germination, hindered growth, and early leaf senescence, which collectively cause significant yield losses (Anjum et al., 2017; Kou et al., 2022). These changes are particularly critical during anthesis, followed by other key growth stages such as grain filling, booting, and tillering, with drought stress at these stages causing up to 69% yield losses in wheat (Khan et al., 2023). Similar negative effects, including reduced photosynthesis, altered stomatal function, and accelerated senescence, have been reported for barley (Liang et al., 2002; Yang and Zhang, 2006). During critical vegetative stages (i.e., tillering and jointing) drought stress reduces the number of fertile tillers, impairs stem elongation, and limits resource allocation, ultimately compromising yield potential (Oğuz et al., 2022). However, drought-tolerant species may exhibit adaptive mechanisms, such as improved water retention and osmotic adjustment, which help mitigate these effects and preserve growth and yield potential (Ghorbanpour et al., 2020).

While significant advancements have been made in understanding how above-ground plant parts adapt to changing climatic conditions, the below-ground response, particularly root system traits under drought stress, remains understudied (Moran et al., 2017; Li et al., 2021a). Enhanced root system functionality is often deemed crucial for achieving a second Green Revolution (Lynch, 2007; Kou et al., 2022). A meta-analysis of field studies indicates that drought stress reduces root length and density while increasing root diameter and root-to-shoot biomass ratio, possibly due to enhanced translocation of photosynthates to roots (Bo et al., 2015; Zhou et al., 2018a; Chen et al., 2022).

Beyond structural changes, drought stress disrupts many plant physiological processes, leading to reduced stomatal conductance, decreased water permeability through cellular membrane, and impaired photosynthesis due to elevated abscisic acid (ABA) concentrations (Liang et al., 2002; Chaves et al., 2003; Li et al., 2021b). Importantly, recovery from drought, a phenomenon influenced by stomatal reopening, leaf water potential restoration, and other factors, often varies with the frequency and severity of drought-rehydration cycles (Talamè et al., 2007; Sicher et al., 2012). This process can induce a “priming” effect, where an initial stress event triggers physiological and molecular adaptations that enhance tolerance to subsequent drought episodes (Kambona et al., 2023). While a single drought/rehydration cycle has demonstrated compensatory potential in mitigating drought-induced damage by restoring plant growth and metabolic functionality (Paul et al., 2023), and recent research has precisely characterized the physiological response to a single drought event in barley (Paul et al., 2024), the impacts of this priming through repeated drought/rehydration cycles on the physiology, water productivity, and long-term growth performance of barley remain unexplored, particularly the underlying physiological mechanisms beyond the recently described morphological re-tillering response (Soleimani et al., 2025).

This gap is particularly critical when considering the foundational nature of vegetative stages, which are decisive for early development and subsequent crop productivity (Farkas and Varga 2025). Although the respond to single drought events is documented for barley across all growth stages (Paul et al., 2023, 2024), the specific acclimation to recurrent drought-rehydration cycles, which are hallmark of climate change, during these formative vegetative stages (e.g., tillering and jointing) remains almost entirely unknown. Since these stages are crucial for setting the potential number of fertile tillers and the root system architecture (Paul et al., 2023; Soleimani et al., 2025), understanding how they respond to repeated stress cycles is essential to predicting and improving crop productivity under fluctuating water availability.

This study aims to address the gap by investigating the physiological responses of barley to multiple drought-rehydration cycles and their influence on growth performance and interval-specific water use efficiency (WUEn). Specifically, we hypothesize that barley plants subjected to intermittent drought conditions, compared to those under continuous drought stress and non-stressed controls, will demonstrate enhanced physiological recovery and improved growth characteristics upon rehydration, mediated by coordinated biochemical regulation. Through this study, we aim to uncover potential drought resistance mechanisms that contribute to barley’s adaptive capacity, thereby providing critical insights toward mitigating agricultural challenges under the looming threat of global climate change.

## 2 Materials and methods

### 2.1 Growth conditions and plant material

The experiment was conducted under controlled glasshouse conditions at BOKU University, Austria, within the UFT facilities located in Tulln an der Donau (48°20′N, 16°03′E). The glasshouse environment was automatically regulated to maintain stable humidity levels (60-75%), temperatures ranging between 18 °C and 26 °C, and a constant light intensity of 1100 μmol m^-^² s^-1^ under a 16-h day/8-h night photoperiod.

The experimental units were pots with dimensions of 12 cm in diameter and 20 cm in height. Each pot was filled with 1,750 g of a commercial potting mix procured from Empfinger Rindenmulch GmbH (Vienna). The growth medium was composed of physically processed fresh wood fibers, quality compost (grade A+), bark humus, sand, clay, rock flour, and an organic NPK fertilizer (initial pH: 6.9). To minimize water loss via soil evaporation, a 100 g layer of coarse sand was uniformly applied to the surface of each pot after sowing.

Following sowing, an initial irrigation was applied to achieve 80% of the soil water-holding capacity (SWHC) across all pots. This moisture level was maintained for six days to promote uniform seedling emergence. Five seeds of the high-yielding spring barley (*Hordeum vulgare* L.) cultivar ‘Elektra HG’, obtained from Probstdorfer Saatzucht GesmbH & Co KG (Probstdorf, Austria), were sown per pot providing adequate plant material for subsequent growth, physiological, and water-use efficiency evaluations. Upon germination, seedlings were thinned to three per pot maintaining consistent plant density and optimal resource allocation. To ensure sufficient plant material for all analyses, two parallel sets of experiments were conducted. The first set was used for morphological and physiological measurements, including biomass and WUEn. The second set was used exclusively for hormonal analyses (ABA and proline in roots and shoots). Each set contained three replicates per treatment.

The pots were arranged in a completely randomised design (CRD) with mono-factorial layout and periodically repositioned during the experiment for uniform exposure to light and temperature conditions. Additionally, pest control was conducted using blue and yellow adhesive sticky traps to effectively monitor and manage insect infestations.

### 2.2 Experimental design and treatments

**Figure 1:**
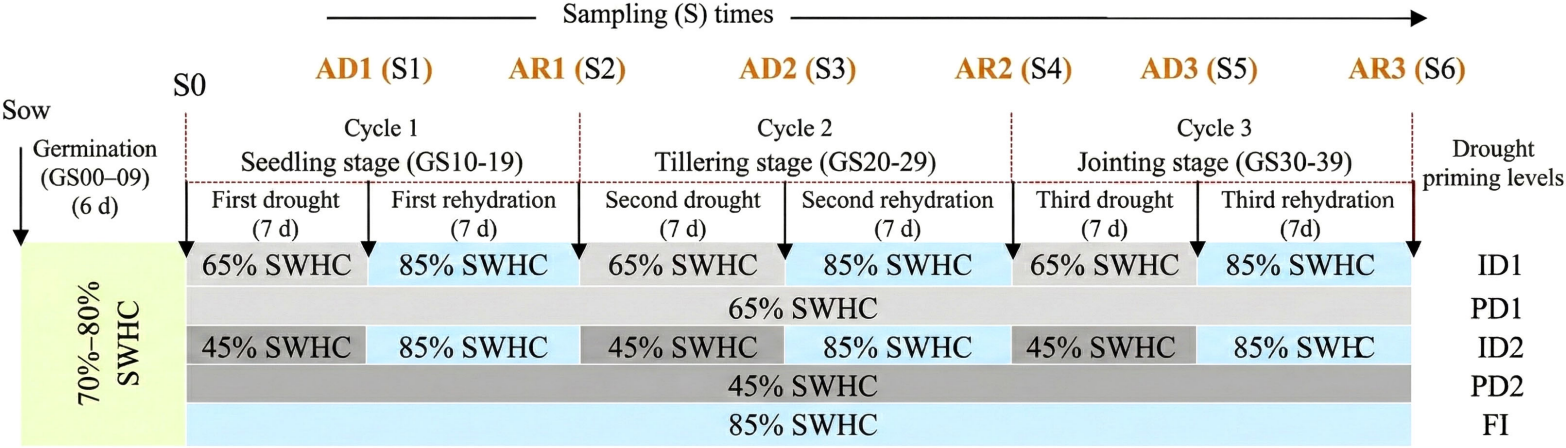
Depiction outlining the water management regimes employed in the study. FI (Full Irrigation): Soil moisture maintained at 85% of soil water holding capacity (SWHC) throughout the experimental period; ID (Intermittent Drought): ID1 and ID2 represent mild (65% SWHC) and severe (45% SWHC) drought conditions, respectively, with alternating cycles of drought and rehydration (rehydration to 85% SWHC); PD (Persistent Drought): PD1 and PD2 represent unaltered mild (65% SWHC) and severe (45% SWHC) drought conditions, respectively, maintained throughout the experimental period without any rehydration; S0, pre-treatment sampling; AD (After Drought): AD1, AD2, and AD3 denote post- drought conditions following the first, second and third drought cycle; AR (After Rehydration): AR1, AR2, and AR3 denote post-rehydration conditions following the first, second and third rehydration cycle, respectively.

The study applied two levels of drought stress, defined by soil moisture thresholds: mild drought (D1), with soil moisture maintained at 65% soil water-holding capacity (SWHC), and severe drought (D2), maintained at 45% SWHC. Each drought level was implemented under two water management regimes: intermittent drought (ID), featuring alternating cycles of drought and rehydration, and persistent drought (PD), characterized by continuous water restriction without rehydration. This experimental structure yielded four drought treatments: mild intermittent drought (ID1), mild persistent drought (PD1), severe intermittent drought (ID2), and severe persistent drought (PD2). In addition, a fully irrigated control group (FI) was sustained throughout the experiment, maintaining a higher soil moisture level of 85% SWHC. These water management strategies are schematically presented in **Figure 1**.

Under the intermittent drought (ID) regime, three cycles of alternating drought and rehydration were imposed during critical vegetative stages, as determined by the Zadoks development scale (i.e., growth stage – GS) for morphological development in barley plants (Lancashire et al., 1991). Each growth stage underwent identical drought/rehydration cycles (7 days drought followed by 7 days rehydration), deferring only in stress intensity and developmental phase: seedling stage (GS 10-19) including sampling times S1, S2, tillering stage (GS 20-29) including S3, S4 and jointing stage (GS 30-39) including S5, S6. In contrast, persistent drought (PD) treatments involved continuous soil moisture restriction at the same stress intensities, 65% SWHC (PD1) and 45% SWHC (PD2), throughout these growth stages (seedling: GS 10-19, tillering: GS 20-29, jointing: GS 30-39), with no rehydration applied.

The experimental design comprised three replicates per treatment, resulting in 90 experimental units (3 replicates × 5 treatments × 6 sampling times). Soil moisture levels were meticulously controlled and monitored daily to ensure consistency with the prescribed treatment protocols. Pots were weighed and irrigated every two days between 18:00 and 19:00 to maintain the target SWHC levels.

### 2.3 Plant sampling

Plant sampling was systematically performed across six distinct dates, each aligned with the completion of a specific drought or rehydration cycle. These phases were categorized to reflect the plants’ response to alternating stress and recovery periods: post-first drought (AD1), post-first rehydration (AR1), post-second drought (AD2), post-second rehydration (AR2), post-third drought (AD3), and post-third rehydration (AR3). During each sampling time (S1-S6), three plants were harvested from each replicate pot. These selected plants were subsequently prepared for downstream analyses, enabling an in-depth exploration of physiological and structural responses to the imposed drought and recovery conditions.

### 2.4 Growth parameters and root morphological analysis

At each sampling time, several growth characteristics were evaluated to characterize plant growth dynamics and biomass allocation. Plant height, measured as the distance from the base of the plant to the apex of the main stem, and visible root length were recorded immediately after harvest. Harvesting plant roots from pots without washing and minimizing damage was accomplished through careful manual handpicking and sieving (Fahey et al., 2017). To assess the dry weight of each sample, the plant material was oven-dried at 75°C for 24 hours, allowing for the calculation of total biomass as the aggregated dry weight of both shoot and root fractions. In order to evaluate the limitations of pre-drought stress on plant growth performance, we calculated Pre-Drought Limitation (PDL) using total biomass measurements across all treatments and vegetative stages. PDL was determined as the percentage difference in mean biomass between stressed treatments and fully irrigated controls (FI), calculated for each vegetative stage in six sampling times (S1-S6) using the formula (Equation 1) (Xu et al., 2009):

(1) PDL(%):BMC–BMT/BMC×100

Where, BMC is the total biomass of fully irrigated control plants, and BMT is the total biomass of drought-stressed plants (ID1, ID2, PD1, PD2). Positive PDL values indicated growth limitation under drought stress, while negative values reflected overcompensation. For analytical purposes, we defined three response categories (color-coding values): PDL > +20% (indicating significant biomass reduction, red), values between -20% and +20% (-20% ≤ PDL ≤ +20%) (neutral/mild response, yellow), and PDL< -20% (stress-induced biomass increase, green). Following the classification of individual responses via PDL, the aggregate effect of drought stress on biomass production was evaluated using a Drought Index (DI). The DI was calculated as the ratio of total biomass under drought (ID1, ID2, PD1, PD2) to well-watered (FI) conditions (DI: BMT/BMC), modified from Abid et al. (2018).

An in-depth analysis of root morphological attributes was undertaken to quantify their role in adaptive responses to drought stress. One intact root sample per experimental unit was immediately preserved at 4 °C to maintain its structural integrity. These samples were later scanned in transparent trays utilizing the WinRHIZO software (Win RHIZO pro 2013 a, Regent Instruments Canada Inc.), enabling the precise measurement of root length and volume. This methodological approach ensures robust data collection and aligns with established protocols for root morphological studies (Pandey et al., 2017).

### 2.5 Measurement of interval-specific water use efficiency (WUEn)

Water use efficiency (WUE) typically refers to the plant growth output (e.g., carbon assimilation, biomass, or productivity) per unit of water input in plants (Aukour and Bani Hani, 2022). Due to the experiment’s sequential sampling design, WUEn was calculated for each interval (S_n>-1_→S_n_, where n = 1-6) between consecutive sampling times (from pre-treatment baseline S0 to S6) (**Figure 1**) as the ratio of biomass gain (B_n_ - B_n-1_) to water consumed during the corresponding interval (ΔW_n_) (Equation 2).

(2) $$\text{WUE}n=\left(B_{\text{n}}-B_{n-1}\right)/\Delta {\text{W}}_{n}$$

Where, n represents interval index (1 ≤ n ≤ 6), B_n_ and B_n-1_ are biomass (g) at sampling times S_n_ and S_n-1_, respectively, and ΔW_n_ is water consumed (mL) between S_n-1_ and S_n_. Due to the coarse sand layer on top of the soil, we assume that evaporation is negligible and all the applied water was transpired.

### 2.6 Measurement of stomatal conductance (*g_s_*)

At six sampling times (S1: AD1, S2: AR1, S3: AD2, S4: AR2, S5: AD3, and S6: AR3; **Figure 1**) stomatal conductance (*g_s_*) to water vapor was measured (in mol H_2_O m^-^² s^-^¹) to quantify leaf-level gas exchange (Busch et al., 2024). The measurement was carried out by a portable porometer (LI 600, Li-Cor Biosciences, Lincoln, NE, USA). For each treatment, five readings were taken from randomly selected leaves and averaged for analysis.

### 2.7 Measurements of stress-responsive (ABA, Proline) metabolites

Frozen leaf and root samples (100 mg fresh weight, n = 15 per tissue) were weighed (Cubis MCE analytical balance, Sartorius) into 2 mL Eppendorf tubes after milling in a 50 mL steel beaker using a Retsch mill (30 Hz, 30 s) under liquid nitrogen. Samples were extracted with 1 mL of extraction solution A (organic solvent/water, 3:1 v/v + 0.1% formic acid; organic solvent = methanol/acetonitrile, 1:1 v/v) for 15 min in an ultrasonic bath, followed by centrifugation at 18,000 × g. A 200 µL aliquot of the supernatant was transferred to an HPLC vial with insert and diluted with 100 µL of solution B (water + 0.1% formic acid) (Doppler et al., 2022).

Proline and ABA were analyzed using a Vanquish Horizon HPLC system (Thermo Fisher Scientific) coupled to an Orbitrap IQ-X mass spectrometer equipped with a heated electrospray ionization (ESI) source. Separation was achieved on a Waters Acquity UPLC HSS T3 column (2.1 × 150 mm, 1.8 µm particle size) with a flow rate of 0.3 mL min^-1^. Column oven was operated at a temperature of 35°C and the autosampler was cooled to 10°C). The mobile phase consisted of water (solvent A) and methanol (solvent B), both with 0.1% formic acid, using the following gradient: 0–0.5 min, 0% B; 0.5–2 min linear gradient to 100% B; 2–3 min, 100% B; 3–5 min, re-equilibration at 0% B. Orbitrap IQ-X was operated in fast polarity switching mode to acquire full scan mass spectra, with a scan range of *m/z* 105 to 270 and a mass resolution of 90,000 full width at half maximum (FWHM) at *m/z* 200. Spray voltage was set to 2950 V for positive and 1950 V for negative ionization mode. Flow rates of sheath and auxiliary gas were set to 45 and 10 arbitrary units, respectively, and the vaporizer temperature to 290°C.

### 2.8 Data analysis

All recorded data were analysed statistically using R software (version 4.4.1). To test for significant differences among treatment means for individual traits, one-way analysis of variance (ANOVA) was performed using R Stats package. Where appropriate (p ≤ 0.05 for F-values), *post hoc* comparisons were conducted using Duncan’s multiple range test (DMRT) to identify specific treatment differences.

To complement this analysis and identify major patterns of variation across all measured traits simultaneously, Principal Component Analysis (PCA) was performed using the FactoMineR package (version 2.12) in R. A separate PCA was run for each of the six sampling times (S1-S6) using the original, replicate-level data (n = 15 per sampling time: 5 treatments × 3 replicates). The analysis was based on a correlation matrix, with all variables centered and scaled to unit variance prior to analysis. The 95% confidence ellipses in the scores plots were derived from the distribution of these replicates. All visualizations, including scores and loadings plots, were generated using the factoextra package (version 1.0.7).

The dataset for sampling times (S1- S5) included morphological traits (RV: root volume, RDW and SDW: root and shoot dry weight, respectively, PH: plant height), and physiological traits (WUEn, *g_s_*). For the final sampling time (S6), hormonal (ABA-r and ABA-s: ABA in roots and shoots, respectively) and osmotic (Pro-r and Pro-s: proline in roots and shoots, respectively) compounds were included alongside the morphological and physiological traits. For each PCA, the first two principal components (PC1 and PC2) were retained for biological interpretation based on the scree plot criterion and their cumulative percentage of explained variance.

## 3 Results

### 3.1 Morphophysiological traits across different growth stages

As presented in **Supplementary Table 1** (SI, section 2), analysis of variance (ANOVA) revealed significant effects of drought treatments on several morpho-physiological traits in barley plants. The highest levels of significance (p< 0.01) were observed for root volume (S1 – S6), root dry weight (S1, S2, S6), and shoot dry weight (S3, S5, S6) during the seedling, tillering and jointing stages. Water use efficiency for each interval (WUEn) calculated using Equation 2 exhibited highly significant sensitivity at S1, S3, S4, and S6 (p< 0.01). Moreover, stomatal conductance (*g_s_*), derived from Equation 1, and total biomass showed broadly similar, statistically significant (p< 0.01 or p< 0.05) responses to drought stress treatments at S1, S3, and S6. Except for the early tillering (p< 0.01) and early jointing (p< 0.01) samplings, plant height did not differ significantly among treatments.

#### 3.1.1 Plant height

Statistical analysis revealed no significant differences in plant height (PH) among treatments at the seedling (S1, S2), late tillering (S4), and late jointing (S6) stages (**Supplementary Table S1**). Although all treatments exhibited an overall increase in PH from the seedling (S1–S2) to the jointing (S5–S6) stages, growth patterns and final heights varied significantly at the early tillering (S3) and early jointing (S5) stages (**Supplementary Figure S1a**, **S4**).

The ID1 treatment showed a distinct non-linear response to cyclic drought and rehydration. Its PH stabilized after the second rehydration (S3–S4: 82.00 cm) but decreased after the third drought period (S5: 79.67 cm) (**Supplementary Figure S4**). This was followed by a final, strong recovery after the third rehydration, where PH reached its maximum value (S6: 95.50 cm). In contrast, the ID2 treatment exhibited a pronounced growth surge following the second drought period (S3: 86.17 cm), exceeding the height of FI (77.50 cm) but not that of PD2 (93.17 cm). This surge was followed by a period of stabilization during the second rehydration (S3–S4). However, after achieving the greatest PH among all treatments at S5 (97.17 cm), ID2 decreased by 5.66% following the final rehydration (S6), in contrast to the 19.87% increase observed in ID1.

Unlike the FI control, which showed moderate growth, or the intermittent treatments (ID1, ID2), which stabilized or grew moderately after the second rehydration, the PD2 treatment was the only one to show a reduction in PH (-12.71%) from S3 to S4 (**Supplementary Figure S4**). The PD1 treatment maintained a steadier growth pattern but resulted in a lower PH at S5 (85.67 cm) than the ID2 and PD2 treatments. A detailed, stage-by-stage analysis of PH dynamics is provided in the **Supplementary Information S1** (section 1).

#### 3.1.2 Shoot dry weight (Wt.)

**Figure 2:**
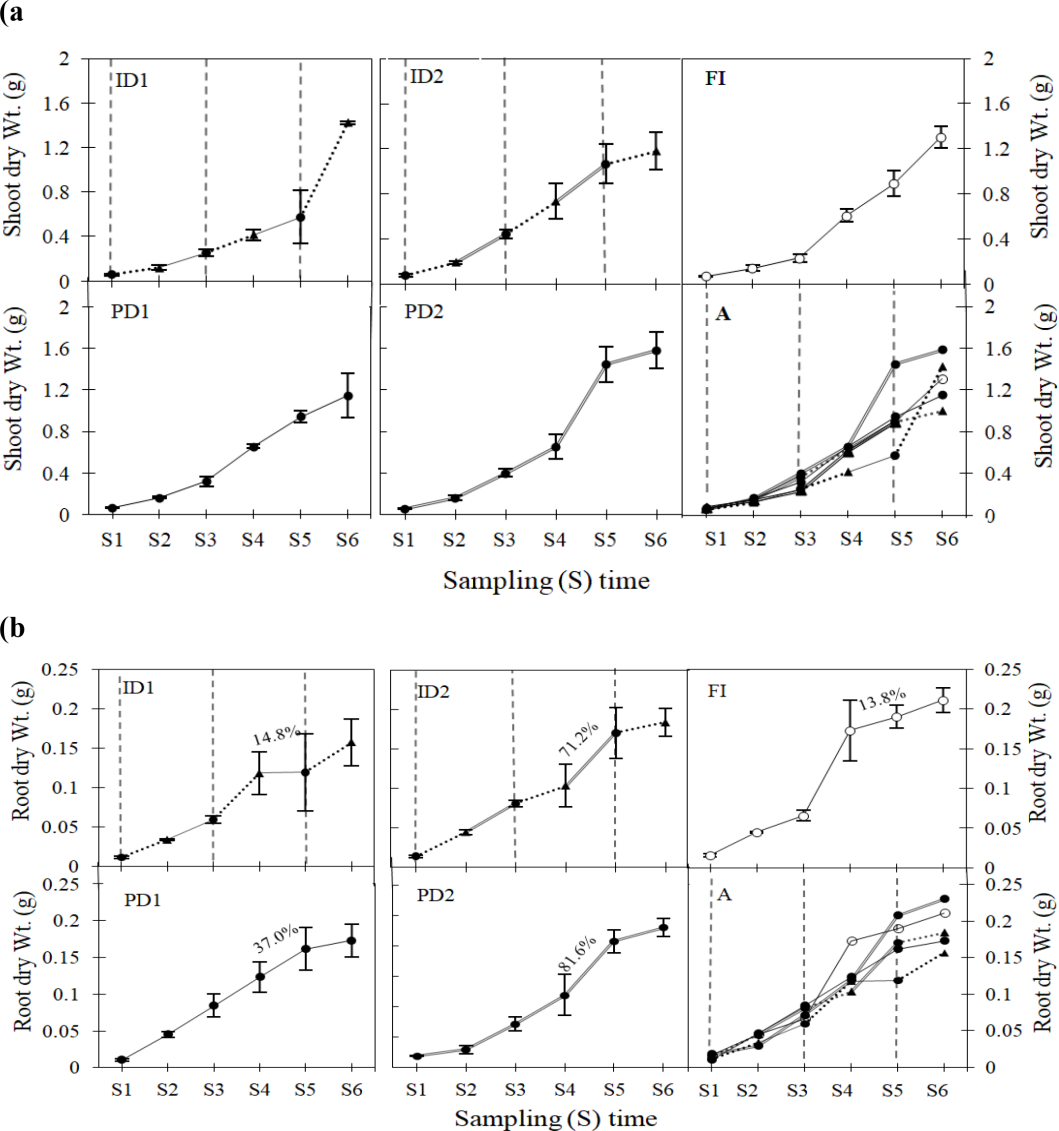
Shoot **(a)** and root **(b)** dry weight (Wt.) of barley plants in response to varying types and levels of drought stress during the vegetative growth stages. Details for FI, ID1, ID2, PD1, and PD2 are provided in **Figure 1**. Percentages represent the increase in root dry Wt. between selected sampling times (S4–S5). Symbols denote the following: well watered control (○, FI), water-deficient plants (●, PD1 and PD2), and rehydrated plants (▲, ID1 and ID2). The vertical dashed line indicates the timing when pots under ID1 and ID2 treatments entered the rehydration period (re-irrigation timing). Dotted lines connect values for rehydrated samples, while single and double black lines represent mild and severe stress thresholds, respectively. Markers represent mean values ± SD (n: 90 plants; 15 plants per sampling (S) time across 6 dates). Seedling stage (S1-S2), Tillering stage (S3-S4), Jointing stage (S5-S6). Image A (in both a, b) shows all treatment trends combined in one graph.

Shoot dry weight did not differ significantly among treatments during the seedling stage (**Supplementary Figure 1b**; for more, see SI-2). A noticeable accumulation of shoot biomass occurred during the transitional phase from seedling to tillering (S2→S3) (**Figure 2a**). By sampling time S3, shoot dry weight under the ID2 (0.365 g) and PD2 (0.401 g) treatments was higher than that of the FI control (0.233 g). The persistent drought treatments also recorded relatively high values at S3 (PD1: 0.316 g; PD2: 0.401 g). During the subsequent rehydration period (S3 to S4), the ID2 treatment demonstrated recovery, with its biomass (0.632 g) reaching a level comparable to FI (0.606 g). By the late tillering stage (S4), differences among treatments were no longer statistically significant (**Supplementary Figure S1b**).

During the jointing stage (S5, S6), the PD2 treatment maintained the highest shoot dry weight among all treatments, reaching 1.582 g at S6. Most notably, the ID1 treatment showed considerable biomass accumulation from S5 to S6, achieving the second- highest value (1.423 g) after PD2 (**Figure 2a**). Conversely, the ID2 treatment showed reduced growth during this period compared to the other treatments.

#### 3.1.3 Root dry weight (Wt.)

Root dry weight was significantly affected by drought treatment across vegetative stages, as detailed in SI-3 (**Figure 2b**, **Supplementary Figure S1c**). At the early seedling stage (S1), root dry weight under the FI and PD2 treatments was significantly greater than that under ID1 and PD1. By the late seedling stage (S2), the root dry weight of PD2 was significantly lower than that of FI, ID2, and PD1. At the early tillering stage (S3), root dry weight in PD1 was significantly greater than in ID1. No significant differences among treatments were detected at the late tillering stage (S4) or the early jointing stage (S5). Furthermore, by the final sampling time (S6), PD2 had a significantly higher root dry weight than ID1, ID2, and PD1, and was statistically equivalent to the well-watered control (FI). The rate of increase in root dry weight during the transitional phase (S4→S5) was in the order of PD2: 81.6% > ID2: 71.2% > PD1: 37% > ID1: 14.8% (**Figure 2b**).

#### 3.1.4 Total dry mass (Biomass)

**Figure 3:**
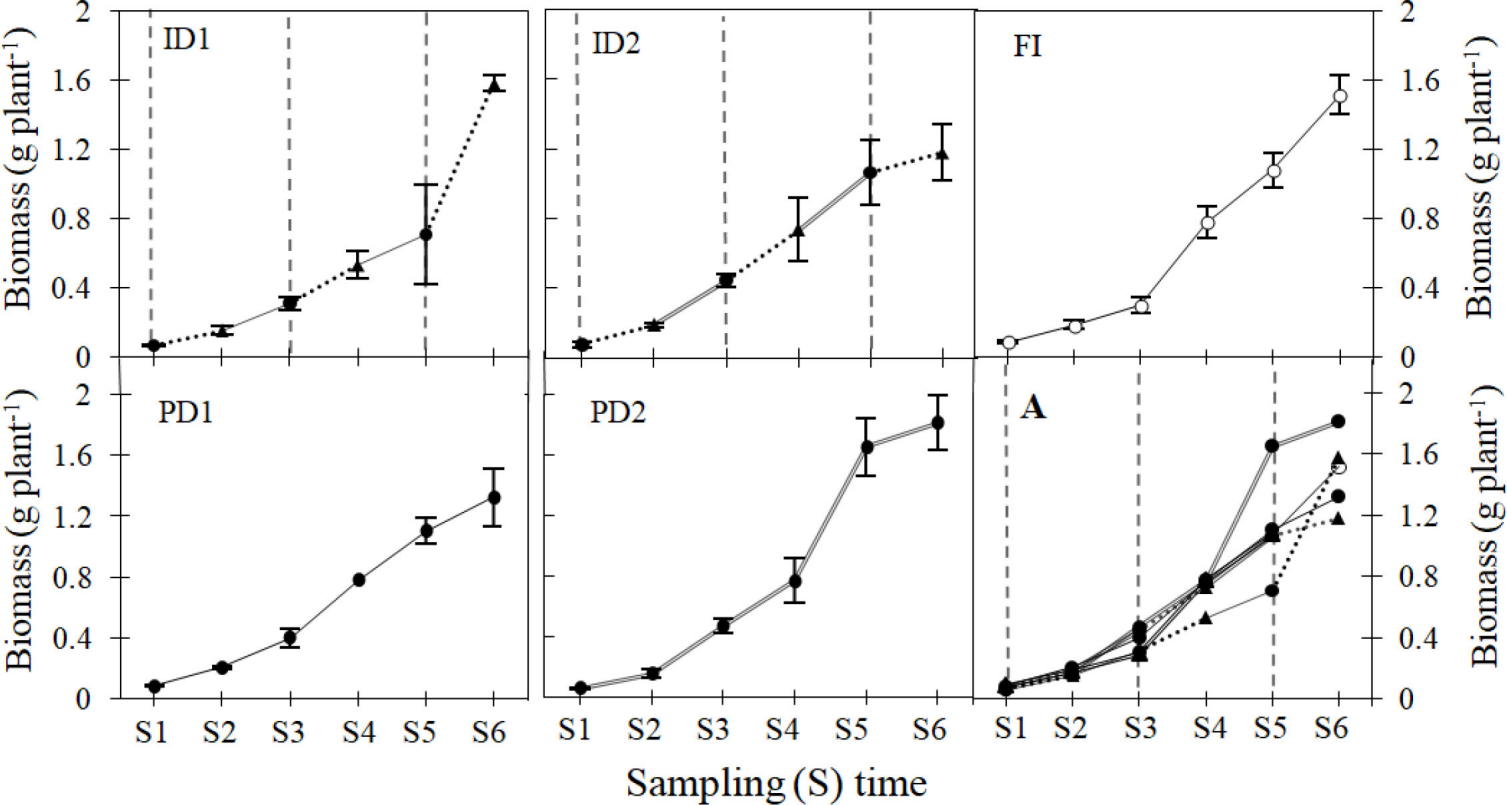
Total dry mass of barley plants in response to varying types and levels of drought stress during the vegetative growth stages. Details of the FI, ID1, ID2, PD1, and PD2 treatments and other information are provided in **Figures 1**, **2**. Seedling stage (S1-S2), Tillering stage (S3-S4), Jointing stage (S5-S6). Image A (in Figure) shows all treatment trends in one graph.

Biomass accumulation was low across all treatments during the seedling stage (S1, S2) (**Figure 3** and **Supplementary Figure S1d**; see SI-4 for a detailed stage-by-stage analysis), and no significant differences were observed at the S2 and S4 sampling times (**Supplementary Table S1**). However, a significant decline in biomass was measured for ID1 (and FI) at S3 (early tillering stage) compared to the other treatments. As plants progressed to later growth stages, differences in biomass accumulation became more pronounced. The highest biomass was recorded in PD2 at S5 (1.654 g plant^-1^). Subsequently, ID1 showed a sharp increase from S5 to S6, which reduced the absolute differences in biomass between ID1 and PD2 by S6. The most notable biomass trends occurred in the PD2 and ID1 treatments. PD2 exhibited significantly higher biomass than ID1 (p< 0.01) at both the S3 and S5 sampling times, with the disparity at S5 being the most substantial. The trends for biomass in these two treatments closely mirrored the patterns observed for shoot dry weight.

#### 3.1.5 Root volume

All treatments showed an increase in root volume over time, with variations depending on drought severity and growth stages, as detailed in SI-5 (**Supplementary Figure S6**). A noticeable increase occurred from S2 to S3 (early tillering stage) compared to the seedling stage (S1-S2) across all treatments (**Supplementary Figure S3**). Notably, in the FI treatment, this increasing trend was sustained at a consistent rate from S2 until the late tillering stage (S4). While all treatments exhibited a steady increasing trend from S3 to S6 (with FI notably from S4), the PD2 treatment displayed a second period of noticeable increase from S4 to S5, distinguishing it from other treatments. This trend aligns with PD2’s peak root volume measured at the jointing stage, with values of 7.94 cm³ (at S5) and 8.82 cm³ (at S6). These values exceeded those of ID2, which had shown dominance during the tillering stage, with root volumes of 4.98 cm³ (at S3) and 5.36 cm³ (at S4) (**Supplementary Figures S1e**, **S6**).

### 3.2 Interval-specific water use efficiency (WUEn)

**Figure 4:**
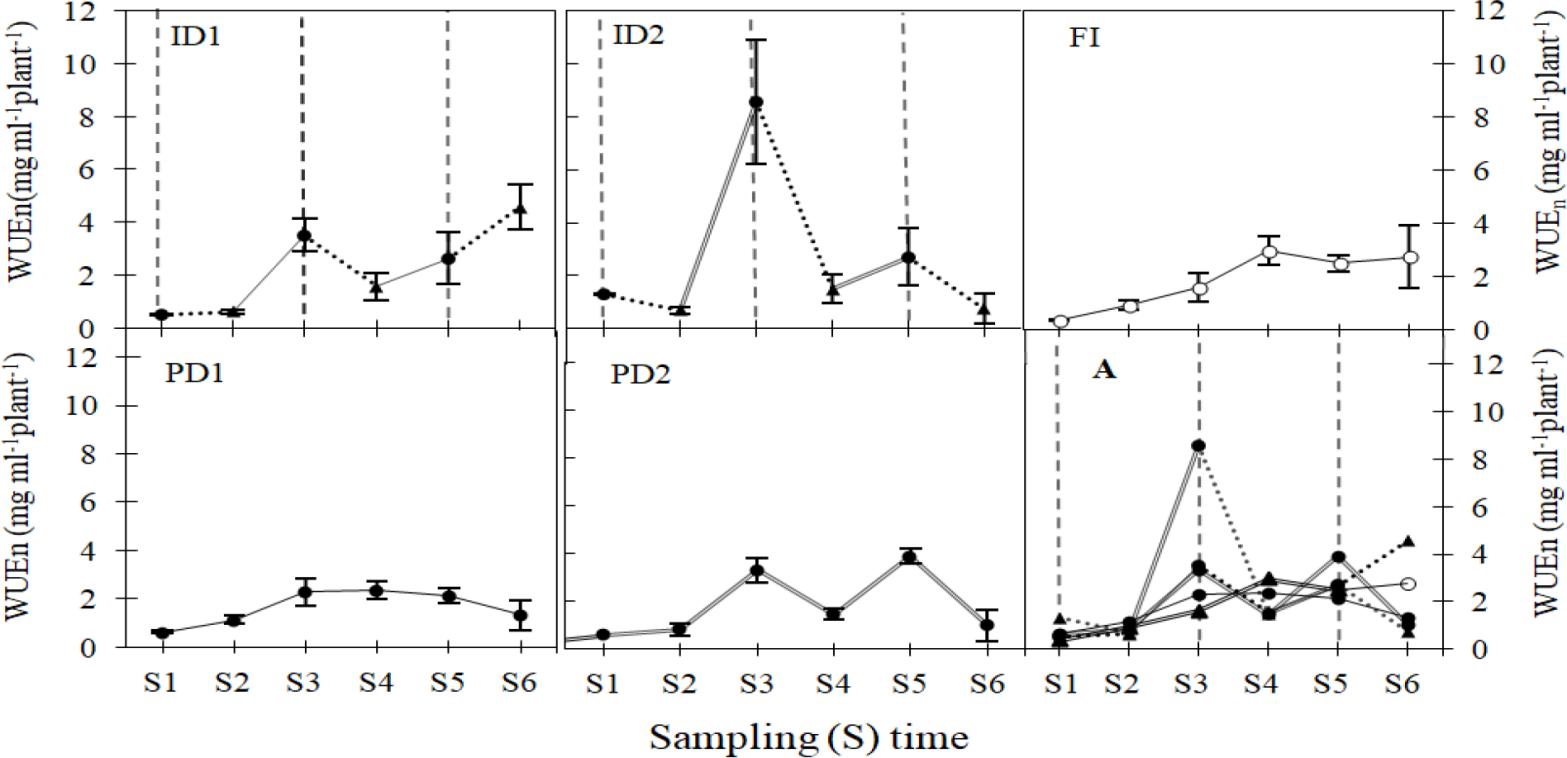
Interval-specific water use efficiency (WUE_n_) of barley plants grown under varying types and levels of drought stress during the vegetative growth stages. Details of the FI, ID1, ID2, PD1, and PD2 treatments and other information are provided in **Figures 1**, **2**. Seedling stage (S1-S2), Tillering stage (S3-S4), Jointing stage (S5-S6). Image A (in Figure) shows all treatment trends in one graph.

The response of WUEn to drought treatment and growth stage is detailed in SI-6 (**Figure 4**, **Supplementary Figure S2a**), as quantified by Equation 2. During the early sampling times (S1, S2), relatively little variation in seedling WUEn was observed across all treatments. The most prominent trend was a notable peak in WUEn under ID2 at S3 (early tillering stage), reaching approximately 8.56 mg ml^-^¹plant^-^¹ as calculated by Equation 2, exceeding all other treatments and sampling times.

WUEn showed a dynamic pattern under severe drought treatments (ID2 and PD2) across each sampling time after the seedling stage (S2 – S6). Specifically, for ID2, WUEn increased during the period from S2 to the tillering stage (S3), reaching 8.56 mg ml^-^¹plant^-^¹, and during the period from S4 to the jointing stage (S5), reaching 2.70 mg ml^-^¹plant^-^¹. However, it displayed a decrease during the period from S3 to S4, reaching 1.48 mg ml^-^¹plant^-^¹, and during the period from S5 to S6, reaching 0.76 mg ml^-^¹plant^-^¹. Similarly, for PD2, WUEn increased during the period from S2 to S3 (reaching 3.33 mg ml^-^¹plant^-^¹) and from S4 to S5 (reaching 3.89 mg ml^-^¹plant^-^¹), but decreased during the period from S3 to S4 (reaching 1.47 mg ml^-^¹plant^-^¹) and from S5 to S6 (reaching 1.01 mg ml^-^¹plant^-^¹).

Under mild drought treatments (ID1, PD1), the WUEn of PD1 exhibited a slight increase from S2 to S4, reaching a maximum of 2.38 mg ml^-^¹plant^-^¹ at S4. It then declined during the period from S4 to S6, reaching a final value of 1.33 mg ml^-^¹plant^-^¹. In contrast, ID1 showed a sharp increase in WUEn from S2 to S3, reaching a peak of 3.52 mg ml^-^¹plant^-^¹. This was followed by a decrease from S3 to S4 (during the tillering stage), reaching 1.57 mg ml^-^¹plant^-^¹. Subsequently, ID1 displayed a sustained increase during the period from the late tillering stage (S4) to the late jointing stage (S6), reaching 4.57 mg ml^-^¹plant^-^¹. This pattern highlights the potential benefits of periodic re-watering during short drought intervals.

### 3.3 Gas exchange

**Figure 5:**
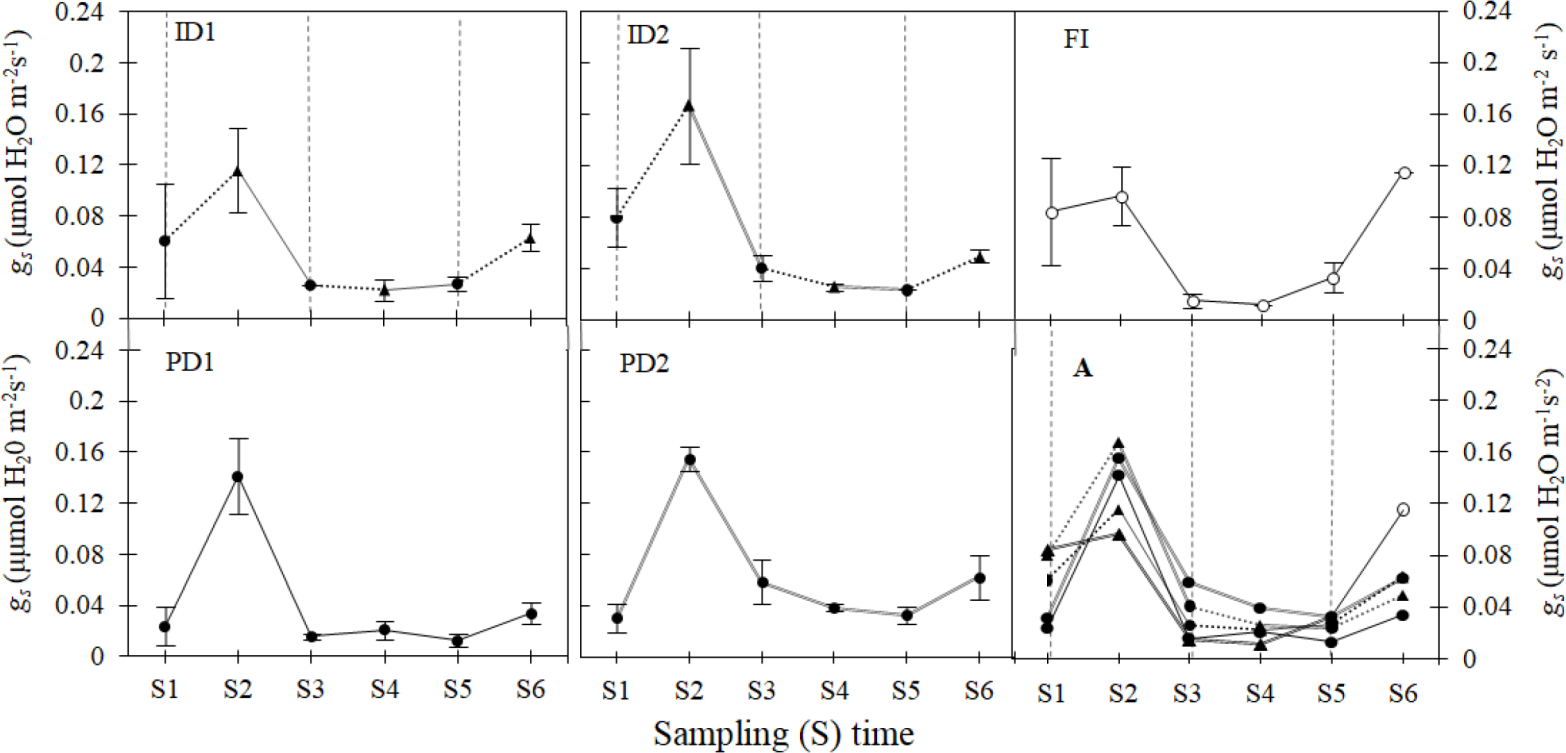
Stomatal conductance (*g_s_*) for water vapour in barley leaves during the vegetative growth stages as affected by varying types and levels of drought stress. Details of the FI, ID1, ID2, PD1, and PD2 treatments and other information are provided in **Figures 1**, **2**. Seedling stage (S1-S2), Tillering stage (S3-S4), Jointing stage (S5-S6). Image A (in Figure) shows all treatment trends in one graph.

Following the initial drought stress at S1, stomatal conductance (*g_s_*) calculated via Equation 1 increased notably from S1 to S2 across all treatments (**Figure 5**, **Supplementary Figure S2b**). At both the S1 and S2 sampling times (seedling stage), no significant differences were observed among the treatments. From S2 to S3, a notable drop in *g_s_* occurred across all treatments. Despite this overall decline, the *g_s_* values measured at S3 were higher in PD2 and ID2 (0.059 µmol m^-1^s^-1^ and 0.041 µmol m^-1^s^-1^, respectively) compared to other treatments. At the S4 sampling time, there was no significant difference observed in *g_s_* among ID2, ID1, and PD1. In contrast, PD2 exhibited a significantly higher *g_s_* value (0.039 µmol m^-1^s^-1^) compared to the other treatments. At the early jointing stage (S5), PD1 had the lowest calculated *g_s_* value (0.013 µmol m^-1^s^-1^), according to Equation 1 followed by ID2 (0.023 µmol m^-1^s^-1^). The highest *g_s_* values were observed in ID1, PD2 and FI, which were statistically at par. At the late jointing stage (S6), aside from FI, the highest *g_s_* values were observed in ID1 (0.063 µmol m^-^² s^-^¹) and PD2 (0.062 µmol m^-^² s^-^¹). The trend observed for *g_s_* mean values from S4 to S6 under the ID1 treatment corresponded well with that of WUEn (**Figure 4**) and biomass (**Figure 3**), but at a lower level.

### 3.4 Proline and ABA metabolites quantification

**Figure 6:**
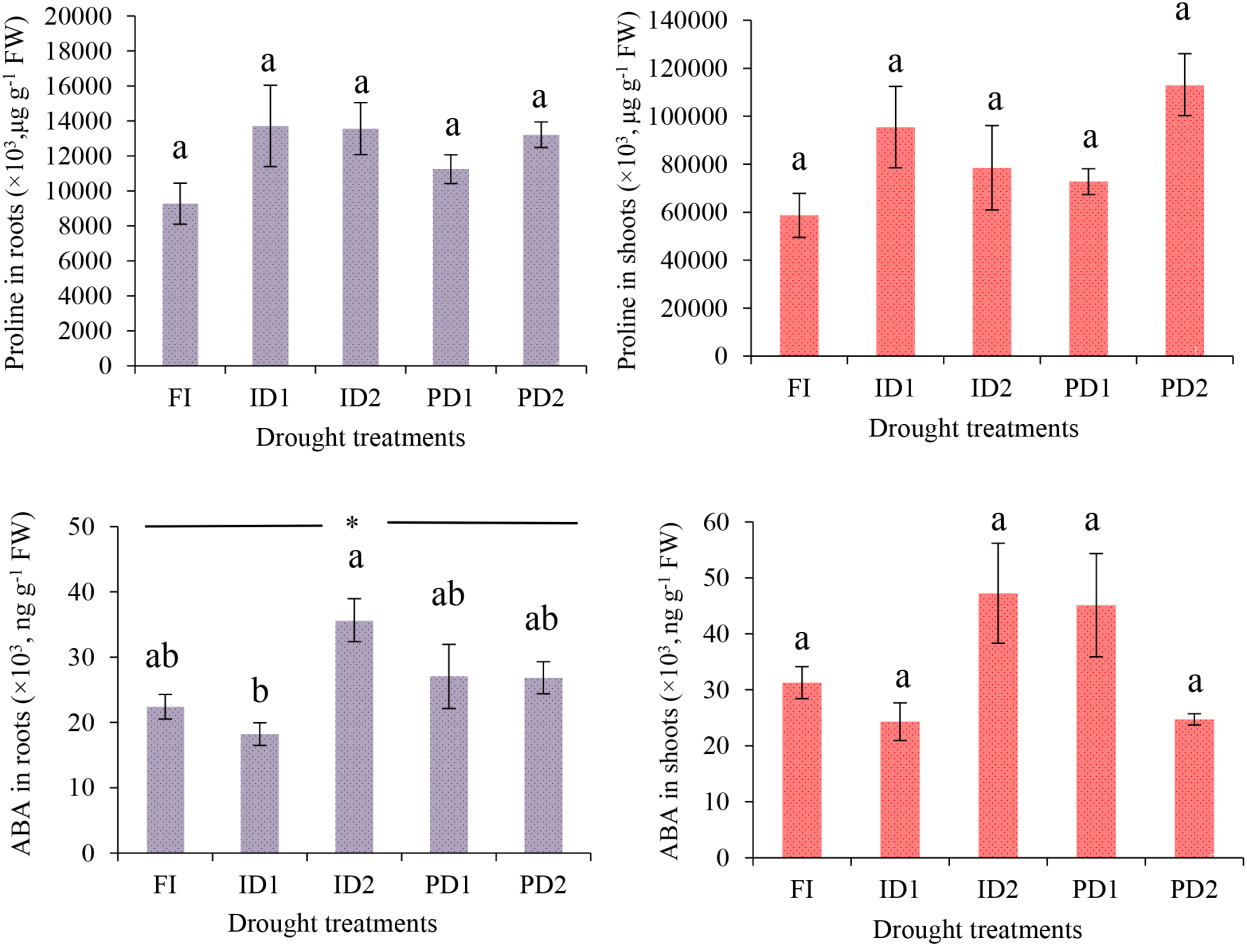
Changes in proline, and abscisic acid (ABA) contents of the leaves and roots exposed to third rehydration cycle (S6-AR3) at late joining stage. Data represent mean ± SD (n = 3). Different letters on the bars indicate significant differences according to Duncan’s multiple range tests (p < 0.05). Details for FI, ID1, ID2, PD1, and PD2 are provided in **Figure 1**; FW, fresh weight, ABA, abscisic acid (stress hormone); Proline (osmoprotectant).

At the late jointing stage (S6), proline accumulation exhibited distinct patterns between roots and shoots under drought stress regimes (**Figure 6**). Shoot proline concentrations were consistently 5–8 times higher than root levels across all treatments. However, neither stress intensity (mild *vs*. severe) nor stress pattern (intermittent *vs*. persistent) had a significant effect on proline levels in either tissue (p > 0.05). In contrast, ABA responses at S6 were organ-dependent, with roots showing greater sensitivity to treatment effects than shoots (**Figure 6**). Root ABA levels varied significantly among treatments (p< 0.01), with the highest accumulation observed in ID2 — 96% greater than in ID1. Both persistent stress treatments (PD1/PD2) exhibited intermediate root ABA levels, with no significant difference between stress intensities (p > 0.05). Control plants (FI) and ID1 roots showed different ABA recovery patterns. Unlike roots, shoot ABA levels remained unaffected by treatment (p > 0.05).

### 3.5 Drought index dynamics across vegetative growth stages

**Figure 7:**
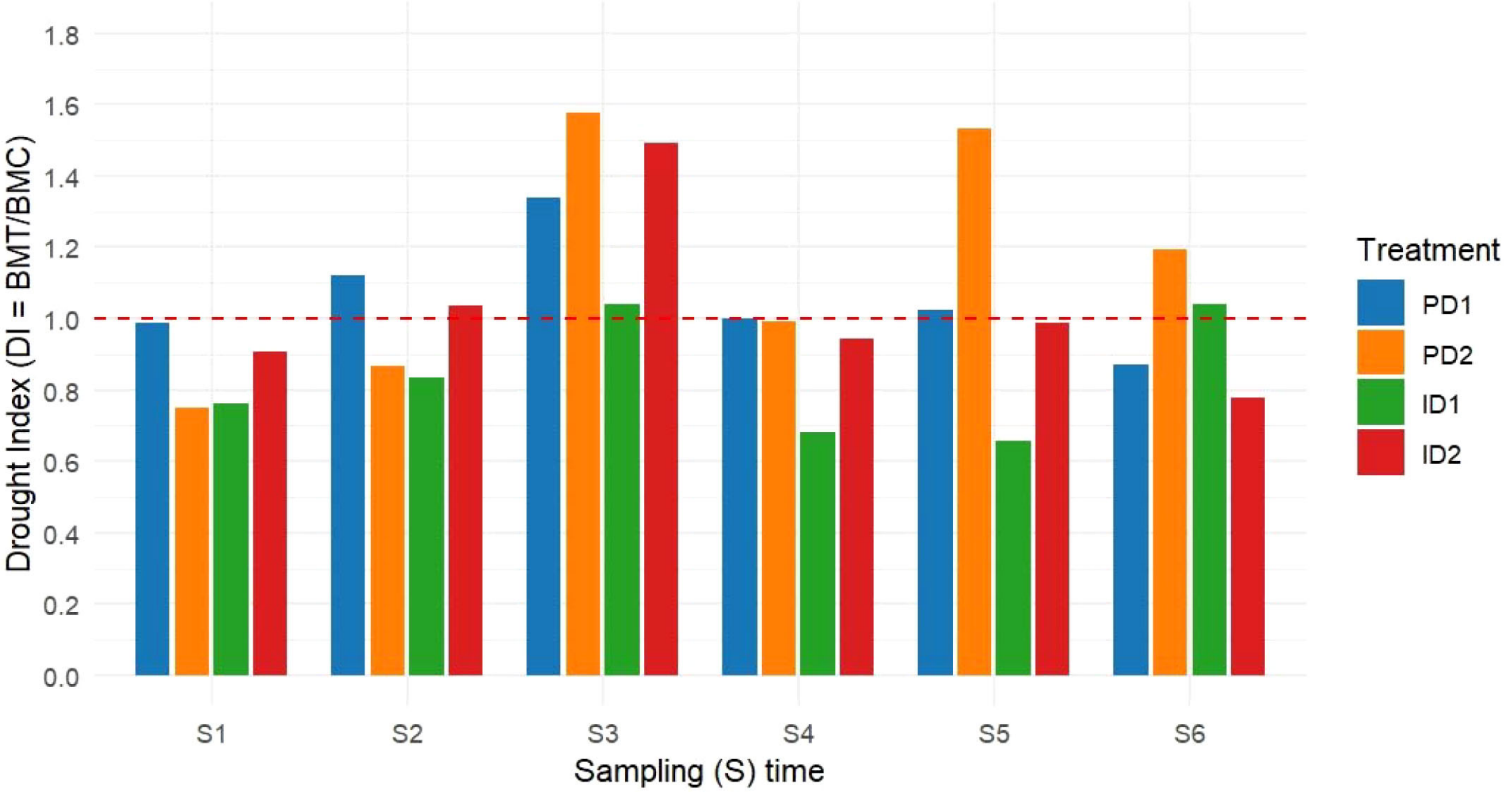
Drought index (DI) dynamics across sampling times and treatments. Bar plot showing DI (BMT/BMC) for each treatment (ID1, ID2, PD1, PD2) at six sampling times (S1–S6). Details for FI, ID1, ID2, PD1, and PD2 are provided in **Figure 1**. DI, BMC, and BMT represent drought index, total biomass of fully irrigated control plants, and the total biomass of drought-stressed plants (ID1, ID2, PD1, PD2), respectively. The red dashed line (DI = 1) indicates the control baseline (biomass of well-watered plants), values > 1 denote overcompensation, and values < 1 indicate drought-induced reduction.

During the seedling stage (S1-S2), the DI values revealed differential responses among treatments (**Figure 7**). ID1 and PD2 treatments exhibited DI values ranging from 0.75 to 0.83, indicating biomass reduction relative to control conditions. In contrast, PD1 maintained DI values near the control baseline (0.99-1.12), while ID2 showed recovery from S1 (DI: 0.906) to S2 (DI: 1.035). The tillering stage (S3-S4) displayed distinct response patterns. At S3, all treatments demonstrated DI values exceeding 1.0, with PD2 reaching 1.577, ID2 at 1.491, PD1 at 1.340, and ID1 at 1.041. By S4, DI values returned toward control levels across most treatments, though ID1 maintained a reduced DI value of 0.683. During the jointing stage (S5-S6), treatment-specific patterns emerged. PD2 showed elevated DI values at both S5 (1.532) and S6 (1.195). ID1 progressed from 0.656 at S5 to 1.042 at S6, while ID2 and PD1 showed final DI values of 0.779 and 0.873, respectively, at S6.

### 3.6 Pre-drought limitation across vegetative growth stages

**Figure 8:**
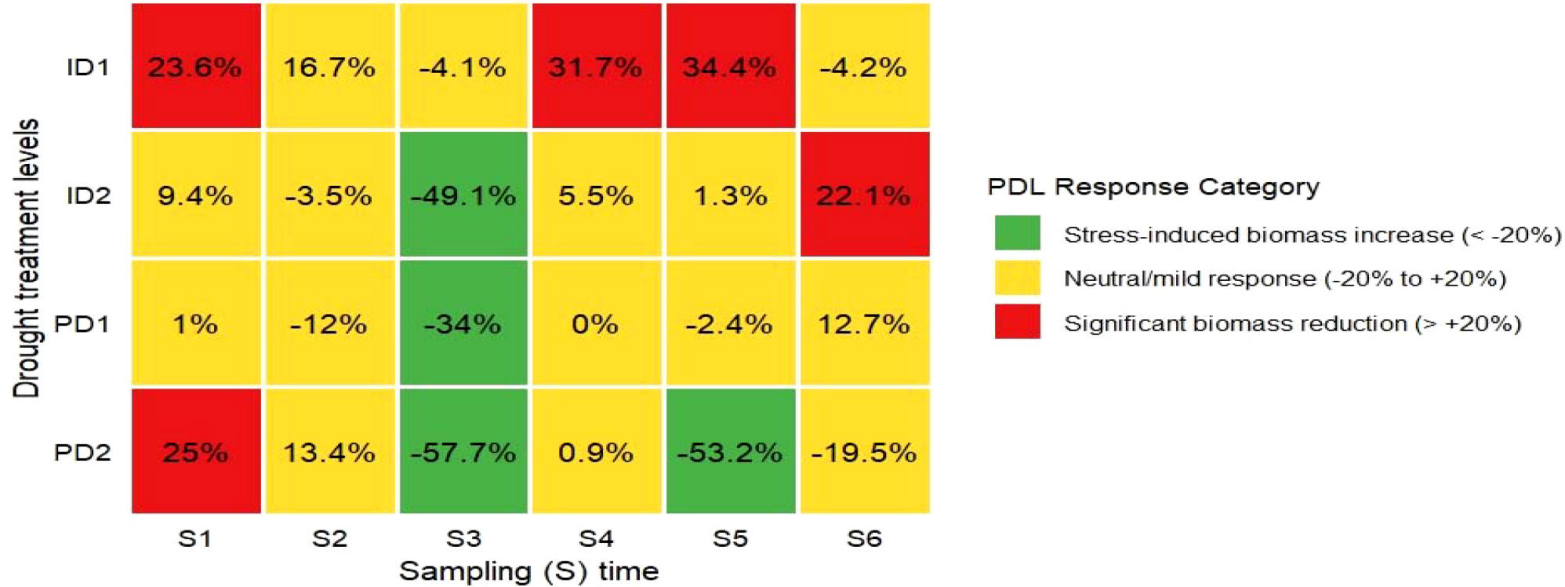
Heatmap of pre-drought limitation (PDL) in barley under different drought treatments (PD1, PD2, ID1, ID2) across six sampling (S1–S6) times. Figure represent percentage changes in biomass relative to control conditions, categorized into three response classes: stress-induced biomass increase (<−20%, green), neutral/mild response (−20% to +20%, yellow), and significant biomass reduction (>+20%, red). Treatments are ordered by drought severity, with PD indicating persistent drought and ID indicating intermittent drought. Details for FI, ID1, ID2, PD1, and PD2 are provided in **Figure 1**.

During the seedling stage (S1-S2), treatments ID1 and PD2 exhibited PDL values of 23.6-25.0% and 13.4-25.0% respectively, indicating significant biomass reduction (**Figure 8**). Treatment PD1 showed neutral responses with PDL values ranging from -12.0% to +1.0%, while ID2 transitioned from +9.4% at S1 to -3.5% at S2. The tillering stage (S3-S4) displayed pronounced treatment variation, with PD2 showing -57.7% PDL at S3, followed by ID2 (-49.1%) and PD1 (-34.0%). By S4, PD1 and PD2 returned to near-neutral levels (+0.03% and +0.9% respectively), while ID1 maintained +31.7% PDL and ID2 showed +5.5%. During jointing stage (S5-S6), PD2 sustained negative PDL values (-53.2% at S5; -19.5% at S6). ID1 progressed from +34.4% at S5 to -4.2% at S6, while ID2 and PD1 concluded with positive PDL values of +22.1% and +12.7% respectively at S6. Treatment PD1 maintained consistent neutral to mild responses across multiple sampling times.

### 3.7 Multivariate analysis of barley’s drought response during vegetative growth stages

**Figure 9:**
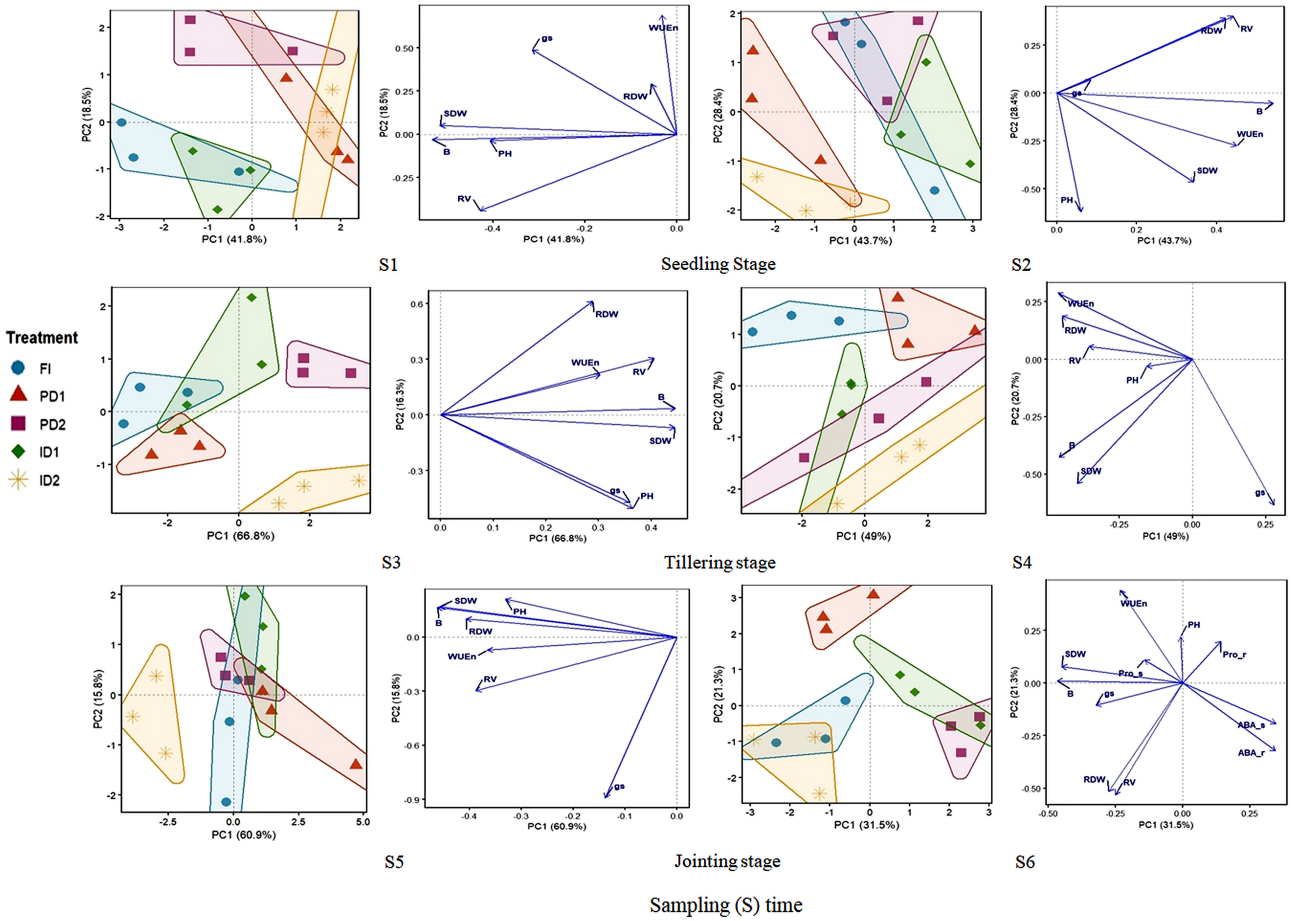
Principal component analysis (PCA) of barley responses to varying types and levels of drought stress during the vegetative growth stages. For each sampling (S) time, the left plot (Scores) displays individual plant responses colored by treatment (FI: Full Irrigation; ID1 and ID2: mild and severe intermittent drought, respectively; PD1 and PD2: mild and severe persistent drought, respectively; for details see **Figure 1**) with 95% confidence ellipses. The right plot (Loadings) shows the contribution of all measured variables to the principal components. The loadings plot is interpreted as follows: the length of each arrow represents the magnitude of a variable's contribution, with longer arrows having a stronger influence on the treatment separation seen in the left plot. The direction of the arrow indicates how variables correlate; variables pointing in the same direction are positively correlated. Consequently, the position of a treatment cluster in the left plot is driven by the variables pointing in that same direction in the right plot. Variables are grouped as: Morphological (SDW: shoot dry weight; RDW: root dry weight; RV: root volume; B: total biomass; PH: plant height, Physiological (gs: stomatal conductance; WUEn: interval-specific water use efficiency), and Hormonal (Pro-r/Pro-s: proline in roots/shoots; ABA-r/ABA-s: ABA in roots/shoots).

Principal component analysis (PCA) was performed for six sampling times (S1-S6) to identify the major patterns of variation in plant traits. The analysis integrated morphological and physiological traits across all stages, with the addition of hormonal (ABA) and osmotic (proline) compounds at the final sampling time (S6). The cumulative variance explained by the first two principal components (PC1 + PC2) was high across all stages, though their individual contributions evolved throughout development (**Figure 9**). PC1 consistently captured the strongest pattern of treatment separation. However, the traits defining PC1 shifted across development: during stages S1-S5 (explaining 41.8-66.8% of variance), it was predominantly associated with PH, shoot dry weight, and total biomass, hereinafter referred to as the ‘Growth and Biomass Axis’. In contrast, at the late jointing stage (S6) (31.5% variance), it was primarily associated with the accumulation of ABA and proline in both roots and shoots, representing a ‘Biochemical Stress Response Axis’. PC2 captured secondary patterns, which were primarily associated with WUEn and *g_s_* for sampling times S1, S3, S4, and S5 (hereinafter defined as ‘Water Relations Axis’), and with root architectural traits at S2 (the ‘Architectural Growth Axis’). Its contribution to total variance ranged from 13.4% to 28.4%.

The PCA biplots revealed distinct clustering patterns among treatments. The primary separation of treatments (known as initial determinant of physiological response) was driven more by stress severity than by the drought pattern (persistent *vs*. intermittent): Across the early and mid-vegetative stages (S1 to S4), the two severe drought treatments (PD2 and ID2) consistently grouped together, while the two mild drought treatments (PD1 and ID1) formed a separate cluster; and the full irrigation (FI) control was distinctly separated from all drought-stressed groups. By the joining stages (S5-S6), the clustering pattern showed greater overlap, coinciding with the introduction of biochemical traits at S6, which were strongly associated with the separation of treatments along the primary axis based on their distinct profiles of hormonal and osmotic stress acclimation.

## 4 Discussion

Drought conditions, whether persistent or intermittent, can trigger a “stress-priming” response in plants characterized by physiological and morphological adaptations including tighter stomatal conductance (*g_s_*) control, enhanced root biomass allocation, and improved water-use efficiency (WUE) (Seleiman et al., 2021; Wang et al., 2023). Our drought index (DI) analysis reveals these priming responses follow distinct developmental-stage patterns, with initial seedling vulnerability (DI< 1 for all treatments) transitioning to robust recovery capacity upon rewatering, particularly at ID2 (1.04 DI at S2) (**Figure 7**). These drought-induced modifications lead to temporarily accelerated growth rates upon rewatering, even surpassing those of well-irrigated plants, as stress-primed plants optimize resource use (Xu et al., 2010; Sicher et al., 2012). Notably, the DI values demonstrate how these acclimatory modifications can confer transient growth advantages upon rehydration, particularly during the tillering phase where all treatments including persistent drought treatments (PD1/PD2) showed DI >1 (S3), with primed plants exhibiting superior growth rates compared to non-stressed controls, a phenomenon attributable to stress-induced optimization of resource acquisition and allocation strategies (Aspinall et al., 1964; Xu et al., 2009; Ke et al., 2025).

Consistent with these priming effects, biomass accumulation in our study followed distinct patterns across treatments. As anticipated during early vegetative growth, biomass accumulation remained low in the seedling stage across all treatments (**Figure 3**, **Supplementary Figure S1d**), reflected in DI values<1 (S1 range: 0.75-0.99) (**Figure 7**). However, as plants progressed to later developmental stages, treatment differences became increasingly pronounced in their biomass trajectories. While PD2 accumulated the most biomass by early jointing stage (S5; DI:1.53) through structural overinvestment in tillers, the notable increase in biomass for ID1 at S6 (DI: 1.04) likely represents post-stress compensatory growth, a well-documented phenomenon where properly primed plants recover and even exceed pre-drought biomass levels following rehydration (Sicher et al., 2012; Ke et al., 2025). This successful recovery in ID1 is further supported by its root ABA profile, which showed complete post-hydration recovery to control levels (**Figure 6**), indicating a resolved stress signal that permitted renewed growth. These compensatory responses, as captured by DI progression from<1 to >1 values, are driven by stress-priming mechanisms, whereby physiological and biochemical adaptations (e.g., enhanced WUEn and optimized resource allocation) facilitate accelerated recovery (Wang et al., 2023; Gao et al., 2024). In the present study, these mechanisms were found more effective under mild intermittent stress (ID1) (S6 DI > 1 *vs*. S5 DI< 1), while severe intermittent stress (ID2) (DI: 0.78 at S6) disrupts the recovery progression (**Figure 7**). The presumable high root ABA in ID2 (96% greater than ID1), even after rewatering at S6 (as seen in **Figure 6**), provides a mechanistic explanation for its impaired recovery, as the persistent ABA signal likely maintained stomatal closure and inhibited growth. The reduction in ID1’s PDL from +34.4% (S5) to −4.25% (S6), further demonstrates its capacity for rapid post-stress recovery (**Figure 8**).

These compensatory growth responses are consistent with findings across multiple species. For instance, Marchetti et al. (2019) used image-based phenotyping to demonstrate that certain barley populations exhibit superior drought recovery upon rehydration during vegetative growth – a pattern our DI values quantified as ID1’s progression from 0.76 (S1) to 1.04 (S6) (**Figure 7**). Similarly, Abid et al. (2018) observed in wheat that post-drought rehydration stimulates physiological and biochemical processes, driving renewed biomass production. Our PDL heatmap corroborates this, showing ID1’s full recovery (−4.25% at S6) versus ID2’s sustained limitation (+22.1%), a disparity strongly linked to their divergent root ABA responses (**Figures 6**, **7**). A meta-analysis by Zhou et al. (2018a) further supports that compensatory biomass responses are a widespread adaptive strategy among plants, enabling rapid recovery after stress relief. Such recovery dynamics suggest that these plants possess highly plastic resource allocation systems, allowing them to optimize growth under fluctuating water availability. In the present study, while PD2’s overcompensation (S6 DI: 1.20) reflects structural adaptation, ID1’s balanced recovery (DI: 1.04) highlights the role of stress-induced plasticity in resource allocation following rewatering (**Figure 1**; Gao et al., 2024). This aligns with the concept that plants dynamically adjust growth patterns, leveraging priming effects and compensatory mechanisms to optimize performance under variable conditions (Ke et al., 2025).

The stage-specific DI patterns in our study further elucidate how developmental timing modulates drought priming efficacy in barley (**Figure 7**). The superior performance of ID1 suggests an optimal stress intensity threshold exists for activating beneficial priming responses without incurring irreversible damage. This treatment’s ability to surpass FI’s biomass (1.580 g *vs*. 1.516 g at S6) through enhanced WUEn (**Supplementary Figures S1**, **S2**) and resource allocation supports Xu et al. (2010) finding that properly modulated stress can improve physiological efficiency beyond well-watered conditions. The DI values quantitatively demonstrate how ID2’s early advantage (S3 DI: 1.49) gives way to limitation (0.78 at S6). This impaired recovery is mechanistically explained by its inability to downregulate the root ABA signal after severe stress cycles (**Figure 6**), which disrupted the priming-recovery progression and ultimately led to a 25.3% biomass reduction relative to ID1 at S6 (**Figure 3**, **Supplementary Figure S1d**). These DI-correlated findings corroborate Zhou et al. (2018a) meta-analysis showing nonlinear declines in compensatory growth potential with increasing stress severity. ID2’s abrupt S3 biomass increase (0.446 g *vs*. FI’s 0.299 g; DI: 1.49) (**Figure 7**, **Supplementary Figure S1d**) suggests that the preceding drought cycle (S1) primed the plants for temporary growth enhancement upon rewatering, likely through ABA-mediated tiller bud activation (**Supplementary Figure S7**). However, this initial response was followed by a progressively slower growth in later vegetative stages (S4 → S6-DI: 0.78) compared to FI, indicating that severe stress ultimately reduced biomass production in ID2 relative to FI, despite the early advantage.

Our multivariate analysis provides a holistic framework for these observations, revealing that the initial physiological impact is governed more by stress intensity than temporal pattern. This is evidenced by the early clustering of severe treatments (PD2, ID2) separate from mild ones (PD1, ID1) (**Figure 9**). During these early stages (S1-S4), the primary axis of variation (PC1) functioned as a ‘Growth and Biomass Axis,’ directly aligning with the initial biomass-driven DI values<1 and the subsequent divergence. Furthermore, the secondary axis (PC2) at the late seedling stage (S2) revealed an ‘Architectural Growth Axis,’ illustrating an early trade-off between vertical shoot growth and root system expansion as a primary morphological response to initial drought stress. This confirms that the core physiological disruption in biomass accumulation and architectural development is first and foremost a function of stress severity.

While the intermittent drought treatments (ID1/ID2) demonstrated barley’s capacity for dynamic recovery responses as quantified by DI fluctuations (**Figure 7**), the persistent drought (PD) treatments revealed a fundamentally different acclimation strategy. Notably, the severe PD treatment (PD2) transiently converged with the performance level of the full irrigation (FI) control during specific developmental stages (S2, S4, and S5), as evidenced by their close clustering in the PCA (**Figure 9**). This convergence reflected a physiological state where PD2 maintained growth-related traits, particularly root and shoot biomass, at levels comparable to FI, despite the persistent water deficit. We propose that the constant water deficit triggered a phased acclimation in PD2 plants: an initial phase of growth maintenance through strategic resource allocation, followed by a late-stage shift toward a stress-optimized phenotype characterized by maximized WUEn under long-term stress conditions. This strategy enhances survival under persistent stress at the expense of maximum growth (Bresta et al., 2018), and stands in clear contrast to the rewatering-driven optimization and damage-repair cycles observed in intermittent drought treatments. The late-stage divergence of PD2 at S6, aligned with ABA and proline accumulation, confirms the distinct nature, and ultimate biochemical cost of this acclimation pathway.

This trade-off between water conservation and carbon gain represents a classic dilemma in C3 cereals like barley, where stomatal regulation must balance CO_2_ uptake for photosynthesis against water loss through transpiration (Kosová et al., 2014). This was particularly critical at the early tillering (S3) and early jointing (S5) stages, where enhanced WUEn directly translated into superior biomass production (see **Supplementary Figure S8**), indicating a superior trade-off between carbon gain and water conservation. Fine-tunes for long-term survival under persistent stress, this acclimated state, contrasts with the ID treatments (especially ID2), where growth was likely hampered by the metabolic cost of repeated damage-repair cycles.

This conceptual framework is supported by their distinct DI trajectories and biomass partitioning: PD1 maintained stable growth (S6 DI: 0.87) through conservative resource allocation, while PD2 achieved vegetative overcompensation (S6 DI: 1.20) at the jointing stage (1.654 g →1.812 g) (**Figure 7**, **Supplementary Figures S1b, d**). Unlike the rehydration-driven compensatory growth seen in ID1 (DI progression to 1.04), PD2’s vegetative overcompensation (1.654g →1.812g at S5-S6) highlights an alternative adaptation pathway where stress-driven tiller development, as evidenced by sustained negative PDL values (−57.7% to −19.6%) and sustained DI>1 (1.58 to 1.20) (**Figures 7**, **8**), is mediated by restricted apical dominance during stem elongation. This shift toward structural overinvestment at the expense of metabolic recovery aligns precisely with Blum (2011) observations of ‘last-resort’ vegetative adaptation in cereals under terminal drought.

The PCA further elucidates this divergence in strategies. As development progressed, a fundamental shift occurred, with PC1 transitioning from a ‘Growth and Biomass Axis’ to a ‘Biochemical Stress Response Axis’ at the late jointing stage (S6), driven by the accumulation of ABA and proline. This shift underscores a strategic reallocation of resources from growth to stress acclimation and explains the increasingly complex treatment clustering in later stages. Within this framework, the PCA trajectories confirm that intermittent drought (ID) fostered a dynamic, plastic response. In contrast, persistent drought (PD) enforced a conservative survival strategy from the early stages, leading to the most intense biochemical stress signaling, which is clearly reflected in their final DI values and biomass outcomes.

The WUEn patterns in barley derived from Equation 2 revealed critical interactions between drought regimes, developmental timing, and biomass partitioning. Early seedling stages (S1–S2) showed minimal WUEn differentiation across treatments (**Figure 4**), consistent with limited biomass accumulation (**Figure 3**) and underdeveloped root systems (root dry weight: FI 0.016–0.045 g; ID1 0.012–0.034 g) (**Figure 2b**), a pattern well-documented in cereals during early vegetative development (Richards, 2006), and reflected in DI values ≤ 1 (**Figure 7**). However, tillering (S3) emerged as a pivotal stage for drought priming, with ID2 achieving the highest WUEn (8.554 mg ml^-1^plant^-1^) as per Equation 2 alongside a pronounced spike in shoot biomass (0.365 g *vs*. FI’s 0.233 g) and corresponding DI of 1.49, demonstrating acute stress priming through physiological adaptations such as tighter stomatal control (0.041 µmol m^-2^ s^-1^; **Figure 5**) driven by its significant root ABA accumulation and rapid shoot recovery (**Figure 2a**) (Tardieu, 2012; Blum, 2017). Yet, this adaptive response proved unsustainable, as ID2’s subsequent root biomass stagnation (0.081 g at S3 → 0.184 g at S6 *vs*. FI’s 0.066 → 0.211 g) coincided with a collapse in WUEn (8.554 µmol m^-2^ s^-1^→ 0.755 µmol m^-2^ s^-1^ by S6) (**Figure 2b**) and declining DI (0.78 at S6), revealing a critical trade-off– transient efficiency gains were achieved at the expenses of long-term resource allocation plasticity, as repeated stress cycles impaired root developmental flexibility (Xu et al., 2009, 2010). Unlike ID2’s short-term optimization, ID1 exemplified balanced recovery: by S6, its superior WUEn (4.572 µmol m^-2^ s^-1^) and total biomass (1.580 g *vs*. FI’s 1.516 g) reflected optimized shoot-root partitioning (shoot: root ratio 7.933 at S6 *vs*. FI’s 6.150; **Supplementary Figure S5**), with moderate root investment (0.157 g) effectively supporting shoot recovery (1.423 g) (**Supplementary Figures S1b, c**). This underscores ID1’s ability to leverage stress priming without destabilizing shoot-root equilibrium, as captured by its stable DI of 1.04, a key distinction from ID2’s imbalance (Wang et al., 2023). Furthermore, proline accumulated primarily in shoots and did not vary with stress intensity, indicating a consistent osmotic adjustment (OA) role across all drought treatments. This implies that the key recovery differences between ID1 and ID2 were driven more by hormonal regulation (ABA) than by changes in osmotic protection strategy. Our results in the cultivated cv. Electra thus support the suggestion by Mejri et al. (2016) that OA, while important in wild barley, is insufficient alone to confer drought tolerance in cultivated species. Furthermore, proline accumulation was specific to shoots and independent of stress intensity (p > 0.05), suggesting that osmotic adjustment played a consistent role across drought treatments (**Figure 6**). This implies that the critical differences in recovery (ID1 *vs*. ID2) were governed more by hormonal regulation (ABA) than by fundamental alterations in osmotic protection mechanisms. Meanwhile, the full irrigation treatment (FI) showed a consistent and progressive increase in WUEn at each vegetative stage, reflecting optimal physiological performance and prolonged photosynthetic activity (Paul et al., 2023) that served as the baseline for DI calculations.

The persistent drought treatments (PD1/PD2) revealed fundamentally divergent adaptation strategies. PD2 exhibited a high-cost adaptation strategy characterized by a jointing-stage WUEn peak (3.891 µmol m^-2^ s^-1^ at S5; **Figure 4**), coinciding with excessive shoot (1.446 g *vs*. FI’s 0.889 g) and root (0.208 g *vs*. FI’s 0.190 g) biomass investment (**Figures 2a, b**) and corresponding DI of 1.53 (**Figure 7**). This resource-intensive response ultimately proved metabolically unsustainable, as evidenced by PD2’s sharp WUEn decline to 1.02 µmol m^-2^ s^-1^ by S6 despite maintaining DI>1 (1.20), a pattern consistent with Blum (2011) model of terminal drought adaptation, where structural overcompensation compromises metabolic resilience. In contrast, PD1 maintained consistently low WUEn (1.330–2.284 µmol m^-2^ s^-1^ after S2) alongside conservative biomass allocation (shoot: root ratio 3.77–6.6 post-S2; **Supplementary Figure S5**) and near-neutral DI (0.87 at S6), supported by its intermediate, stable root ABA level (**Figures 6**, **7**), while PD2’s transient efficiency gains highlighted the physiological costs of unmitigated stress. These trade-offs demonstrate that WUEn dynamics are intrinsically linked to biomass allocation strategies under drought conditions (Liang et al., 2002; Blum, 2009). Notably, where mild intermittent stress (ID1) enabled optimized efficiency and recovery (final DI: 1.04), persistent drought forced plants into dichotomous outcomes: either unsustainable overinvestment (PD2 DI: 1.20) or developmental stagnation (PD1 DI: 0.87).

Stomatal regulation exhibited stage- and treatment-specific patterns. These patterns, as quantified by Equation 1, correlated roughly with DI values, reflecting developmental and drought-adaptive responses (**Figure 5**). A consistent peak in *g_s_* occurred at S2 across all treatments, likely due to improved root-soil contact and heightened carbon demand during early tillering establishment (Carins Murphy et al., 2014). Subsequent *g_s_* decline across all treatments (S3–S5)- including non-stressed FI plants (0.015 → 0.033 μmol m^-^² s^-^¹) – reflects intrinsic developmental programming of water conservation mechanisms through progressive stomatal tightening, independent of drought stress stimuli. The minimal treatment differences in shoot proline and ABA at S6 (p> 0.05) (**Figure 6**) confirm that shoot-level osmotic and hormonal factors were not the primary drivers of the final stomatal patterns; instead, the root-originating ABA signal appears to be the dominant regulator. At S6, treatment-specific divergence emerged, with FI plants achieving peak *g_s_* (0.115 μmol m^-^² s^-^¹) to meet jointing-stage carbon demands (DI = 1). While drought-exposed plants showed partial recovery at S6, they retained significantly suppressed conductance (e.g., PD1: 0.034 μmol m^-^² s^-^¹; 70.4% reduction *vs*. FI) compared to FI. This suppression occurred despite the developmental need for elevated *g_s_* (Li and Wang, 2023; Yang et al., 2023), suggesting ABA-mediated constraints on stomatal reopening capacity in stress-primed plants (**Figures 5**, **6**) that are quantitatively reflected in their DI values.

The observed stomatal responses were closely linked to shifts in biomass partitioning and WUEn, revealing treatment-specific drought adaptation strategies. While PD1 maintained severe *g_s_* suppression (S5: 0.013 μmol m^-^² s^-^¹) with limited biomass accumulation (S6: 1.323 g) and sub-neutral DI (0.87), PD2’s unexpected partial *g_s_* recovery at S6 (0.062 μmol m^-^²s^-^¹) coincided with significant biomass overcompensation (1.812 g; +19.6% *vs*. FI) and elevated DI (1.20) (**Figures 3**, **7**). This suggests PD2’s strategy prioritized structural recovery growth under stress, likely through compensatory tillering (Yang et al., 2023), albeit at the cost of sustained metabolic inefficiency (evidence by low S6 WUEn [1.017 mg ml^-^¹plant^-^¹]). In contrast, ID1 achieved a balance in both stomatal behavior and DI values, with intermediate *g_s_* at S6 (0.063 *vs*. 0.115 μmol m^-^² s^-^¹) and the highest WUEn among drought treatments (4.572 mg ml^-^¹plant^-^¹) corresponding to its optimal DI of 1.04, indicating optimal priming where intermittent mild stress enhanced water conservation without compromising recovery capacity (Blum, 2017; Zhou et al., 2018b).

The divergent *g_s_* trajectories of ID1 and ID2, captured by Equation 1, underscore the role of stress severity and frequency in modulating ABA-mediated stomatal control. ID2’s abrupt *g_s_* collapse post-S2 (0.167 → 0.041 μmol m^-^² s^-^¹) and minimal recovery at S6 (0.0496 μmol m^-^² s^-^¹) reflect cumulative ABA priming under repeated stress (Tardieu and Simonneau, 1998), as directly measured by its significantly elevated root ABA content, which constrained stomatal reopening despite rewatering, likely due to depleted osmotic adjustment capacity (Zivcak et al., 2016). This aligns with ID2’s poor WUEn at S6 (0.755 mg ml^-^¹plant^-^¹), indicating destabilized carbon-water balance under excessive stress. In contrast, ID1’s stable WUEn and intermediate *g_s_* with partial recovery at S6 suggest a threshold effect, where mild stress cycles (ID1) enhanced drought resilience (final DI: 1.04), while intense cycles (ID2) exceeded acclimation capacity. Such threshold-dependent responses are consistent with findings in wheat, where ABA accumulation during early stress stages primes later water conservation (Yoo et al., 2009).

The PCA consistently highlighted a secondary axis (PC2) related to WUEn and *g_s_* — the ‘Water Relations Axis’ — underscoring the pervasive trade-off between carbon gain and water conservation. This axis helps explain the distinct *g_s_* and WUEn trajectories: persistent drought treatments, especially PD1, were strongly associated with this conservation strategy, while intermittent treatments, particularly ID1, occupied a more intermediate position, allowing for flexible stomatal behavior that balanced carbon uptake and water loss, ultimately contributing to its successful recovery and optimal final DI.

The insights from this work, while clarifying the physiological strategies of cv. Electra, must be considered within the framework of our experimental design. Our findings are derived from a single genotype under controlled glasshouse conditions, and thus their broader applicability to diverse genetic backgrounds or field environments remains to be established. Moreover, the physiological patterns observed here, particularly the dynamics of ABA signaling and recovery, raise fundamental questions about the underlying molecular mechanisms that this study was not designed to address.

## 5 Conclusions

In conclusion, our integrated analysis demonstrates that the determinants of drought response in barley are dynamic, shifting from an initial dependence on stress severity to a long-term acclimation shaped by the complex interaction of drought pattern, severity, and developmental stage. This process is characterized by a strategic shift from initial architectural and growth adjustments to sophisticated biochemical mitigation, a key transition clearly captured by the stage-specific evolution of our multivariate analysis.

We provide clear evidence that the early-maturing barley cv. Electra exhibits a high capacity for physiological adaptation to intermittent drought, primarily through ABA-mediated stomatal regulation and compensatory growth upon rehydration. Our findings reveal that mild intermittent drought (ID1) optimizes WUEn and enables full biomass recovery, a process facilitated by the rapid downregulation of root ABA signaling observed following stress relief at S6.

The tillering stage (S3) emerged as a critical developmental window for drought priming, where stress-induced responses significantly influenced later growth and resilience. Moreover, the shoot-specific and stress-intensity-independent proline accumulation indicated that osmotic adjustment played a consistent role across treatments, confirming that differential recovery was governed more by hormonal (ABA) regulation than by osmotic strategy shifts. While persistent drought forced plants into less adaptive survival modes, Electra’s performance under intermittent stress, especially its recovery plasticity and efficiency optimization, highlights its promise as a genetic resource. Therefore, to translate these findings, future work should prioritize multi-genotype field trials and molecular profiling to connect these physiological phenotypes to their genetic determinants and validate their efficacy in semi-arid environments.

## Data Availability

The data presented in the study are deposited in the Zenodo repository, accession number https://doi.org/10.5281/zenodo.17927758.
