# Supplementary material for: Growth responses of spring barley to varying levels of drought priming
Source: Front Plant Sci. 2026 Jan 22;16:1716430. doi: 10.3389/fpls.2025.1716430 (PMC12874091; doi:10.3389/fpls.2025.1716430)
Supplement: Supplementary file 1 [file Supplementaryfile1.docx]

**Supplementary information (SI)**

**Growth Responses of Spring Barley to Varying Levels of Drought Priming**

Zohreh Salehi Soghadi^1^, Peiman Zandi^2,3^, Yaosheng Wang^2^ , Hans-Peter Kaul^1*^

^1^Department of Agricultural Sciences, Institute of Agronomy, University of Natural Resources and Life Sciences, Vienna (BOKU), 3430 Tulln, Austria

^2^ State Key Laboratory of Efficient Utilization of Agricultural Water Resources, Key Laboratory of Dryland Agriculture, Ministry of Agriculture and Rural Affairs of China, Institute of Environment and Sustainable Development in Agriculture, Chinese Academy of Agricultural Sciences, Beijing 100081, China

^3^ Department of Agroecology, Aarhus University, Blichers Allé 20, 8830 Tjele, Denmark

^*^Correspondence should be directed to Hans-Peter Kaul

E-mail: hans-peter.kaul@boku.ac.at

**Section 1**

**SI-1. Plant height (PH)**

Statistical analysis revealed no significant differences in plant height (PH) among treatments at the seedling (S1, S2), late tillering (S4), and late jointing (S6) stages (Table S1). Significant differences were identified at the early tillering (S3) and early jointing (S5) stages (Supplementary Figure S1a). At the early tillering stage (S3), PH varied significantly across treatments. The severe persistent drought treatment (PD2) achieved the greatest height (93.17 cm), followed by ID2 (86.17 cm), ID1 (82.00 cm), and PD1, with the well-watered control FI being the shortest (77.50 cm) (Supplementary Figure S1a). Following the second rehydration (S4), these differences were no longer significant, with all treatments showing similar heights. At the early jointing stage (S5), significant differences re-emerged. The ID2 and PD2 treatments reached the greatest heights (97.17 cm and 94.67 cm, respectively). In contrast, the ID1 treatment showed a decrease in PH to 79.67 cm, resulting in the lowest height among all treatments at this stage. The FI and PD1 treatments displayed intermediate values (86.33 cm and 85.67 cm, respectively). After the final rehydration (S6), no significant differences were observed. The ID1 treatment increased its height by 19.87% to 95.50 cm, while the ID2 treatment decreased by 5.66% to 91.67 cm. The PH of the FI, PD1, and PD2 treatments showed moderate changes, resulting in statistically similar final heights across all treatments.

**SI-2. Shoot dry weight (Wt.)**

Shoot dry weight did not differ significantly among treatments during the seedling stage (S1, S2) (Supplementary Figure S1b). Significant differences emerged at the early tillering (S3) stage, where a significant accumulation of biomass occurred. The PD2 treatment yielded the highest shoot dry weight (0.401 g), followed by ID2 (0.365 g). Both the PD1 (0.316 g) and PD2 treatments recorded higher values than the well-watered FI control (0.233 g). By the late tillering stage (S4), following a rehydration period, these differences were no longer statistically significant. The ID2 treatment demonstrated a strong recovery, with its biomass (0.632 g) reaching a level comparable to FI (0.606 g). During the jointing stage (S5-S6), the PD2 treatment maintained the highest shoot dry weight, reaching a maximum of 1.582 g at S6. The ID1 treatment showed considerable biomass accumulation from S5 to S6, achieving the second-highest value (1.423 g). Conversely, the ID2 treatment showed reduced growth during this final period compared to PD2 and ID1.

**SI-3. Root dry weight (Wt.)**

Root dry weight was significantly affected by drought treatments at specific developmental stages (Supplementary Figure S1c). At the early seedling stage (S1), the FI (0.016 g) and PD2 (0.018 g) treatments had a highly significantly (p < 0.01) greater root dry weight than the ID1 (0.012 g) and PD1 (0.010 g) treatments. By the late seedling stage (S2), this pattern shifted. The root dry weight of the PD2 (0.029 g) treatment was now highly significantly (p < 0.01) lower than that of FI, ID2, and PD1. At the early tillering stage (S3), the PD1 treatment achieved the highest root dry weight (0.085 g), which was significantly greater than that of ID1 (0.059 g). The root dry weight of the ID2 (0.081 g) treatment was also high and statistically similar to PD1, while the FI and PD2 treatments showed intermediate values. During the late tillering (S4) and early jointing (S5) stages, no significant differences in root dry weight were detected among the treatments. At the final jointing stage (S6), significant differences re-emerged. The PD2 treatment had a significantly higher root dry weight than the ID1, ID2, and PD1 treatments and was statistically equivalent to the well-watered FI control. The rate of increase in root dry weight during the S4 to S5 transition was greatest for PD2 (81.6%), followed by ID2 (71.2%), PD1 (37%), and ID1 (14.8%) (Figure 2b).

**SI-4. Total dry mass (Biomass)**

During the seedling stages (S1, S2), biomass accumulation was low across all treatments (Supplementary Figure S1d). At S1 sampling time, FI and PD1 (group a) had significantly higher biomass than ID1 and PD2 (group b). ID2 showed an intermediate value (group ab) that was not statistically different from the highest or lowest groups. By S2, no significant differences remained among the treatments. At the early tillering stage (S3), significant differences emerged. The biomass for the ID1 treatment and the FI control was highly significantly (p < 0.01) lower than that of the PD2, ID2, and PD1 treatments. At the late tillering stage (S4), these differences were no longer statistically significant, with all treatments showing similar biomass accumulation. Pronounced differences re-appeared at the early jointing stage (S5). The PD2 treatment significantly outperformed all others, achieving the highest biomass (1.654 g). The FI, ID2, and PD1 treatments formed an intermediate group (b), while the ID1 treatment had the lowest biomass (group c, 0.708 g), highly significantly less than all other treatments. By the final jointing stage (S6), the PD2 treatment maintained the highest biomass (1.812 g). The ID2 and PD1 treatments resulted in the lowest biomass values at this final stage (1.804 g and 1.323 g, respectively). The trends in biomass for the PD2 and ID1 treatments closely mirrored the patterns observed for shoot dry weight (Figures 2a and 3).

**SI-5. Root volume**

All treatments showed an increase in root volume over time, with significant variations depending on the drought treatment and growth stage (Supplementary Figures S3 and S6). During the seedling stage (S1, S2), the FI treatment consistently maintained a high root volume (S1: 0.23 cm^3^, S2: 0.55 cm^3^), sharing the highest statistical group (a) at S1 with PD1 and at S2 with ID2 and PD1 (Supplementary Figure S1e). The PD2 treatment had the lowest root volume at both S1 (0.14 cm^3^) and S2 (0.34 cm^3^). At the early tillering stage (S3), a significant shift occurred (Supplementary Figure S6). The ID2 treatment achieved the highest root volume (4.98 cm³), significantly greater than FI (2.98 cm^3^) and ID1 (3.02 cm^3^) (Supplementary Figure S1e). At the late tillering stage (S4), the FI treatment demonstrated a strong recovery, attaining the highest root volume (6.07 cm³, group a), which was statistically similar to ID2 (5.36 cm³, group ab). The ID1 treatment had the lowest value (3.82 cm³). During the jointing stage (S5, S6), the PD2 treatment became dominant, achieving the highest root volume at both S5 (7.94 cm³) and S6 (8.82 cm³) and was significantly greater than the ID2, PD1, and ID1 treatments, respectively. The FI treatment maintained a high intermediate value that was not statistically different from the top-performing PD2 treatment. The ID1 treatment resulted in the lowest root volume among all treatments at both S5 (5.02 cm³) and S6 (5.37 cm³). The PD2 treatment's distinctive second period of strong growth (74%) from S4 to S5 distinguished it from the other drought treatments (Supplementary Figures S1e and S6).

**SI-6. Interval-specific water use efficiency (WUEn)**

During the seedling stage (S1, S2), WUEn was relatively low (Supplementary Figure S2a). At S1, ID2 (1.313 mg ml⁻¹ plant⁻¹) was highly significantly (p < 0.01) higher than all other treatments. By S2, PD1 (1.133 mg ml⁻¹ planr⁻¹, group a) achieved the highest value, but was not statistically different from FI and PD2 (group ab). ID1 and ID2 resulted in the lowest WUEn at this stage (group b). At the early tillering stage (S3), a major divergence occurred. The ID2 treatment reached an exceptionally high WUEn (8.554 mg ml⁻¹ plant⁻¹), highly significantly (p < 0.01) outperforming all other treatments. The ID1, PD2, and FI treatments all showed statically similar, lower values. At the late tillering stage (S4), the FI treatment showed the highest WUEn (2.965 mg ml⁻¹ plant⁻¹, group a), which was statistically at par with PD1 (group ab). The severe drought treatments ID2 and PD2 (group c), along with ID1 (group bc), resulted in the lowest values. At the late tillering stage (S4), the FI treatment showed the highest WUEn (2.965 mg ml⁻¹ plant⁻¹, group a), which was statistically similar to PD1 (2.375 mg ml⁻¹ plant⁻¹, group ab). The ID1 treatment showed an intermediate value (group bc), while the severe drought treatments ID2 and PD2 resulted in the lowest values (1.486 mg ml⁻¹ plant⁻¹and 1.470 mg ml⁻¹plant ⁻¹, respectively; groups c). During the jointing stage (S5, S6), the most notable trend was for ID1 (Figure 4 and Supplementary Figure S2a). After an intermediate value at S5 (group a), it achieved the highest WUEn of all treatments at S6 (4.572 mg ml⁻¹plant⁻¹, group a). In contrast, the ID2 and PD2 treatments, which were high performers earlier, declined to the lowest values at S6 (groups c). In contrast, the ID2 and PD2 treatments, which were high performers earlier, declined to the lowest values at S6 (groups c).

**SI-7. Stomatal conductance (*g_s_*)**

During the seedling stage (S1, S2), no significant differences in *g_s_* were observed among treatments (Supplementary Figure S2b). At the early tillering stage (S3), following a notable drop from S2 (Figure 5), significant differences emerged. PD2 achieved the highest *g_s_* (0.059 µmol m⁻² s⁻¹, group 'a'), which was significantly greater than ID2 (0.041 µmol m⁻² s⁻¹), ID1, PD1, and FI. At the late tillering stage (S4), PD2 maintained the highest *g_s_* (0.039 µmol m⁻² s⁻¹, group a), significantly exceeding other treatments. PD1 showed an intermediate value (group bc), which was not statistically different from the ID1/ID2 (group b) or FI (group c). FI resulted in the lowest *g_s_* (0.012 µmol m⁻² s⁻¹).

**Section 2**

**Figure S1.** Biomass and growth responses of barley roots and shoots to mild and severe intermittent and persistent drought stress. Drought treatments were applied to 6-day-old seedlings, subjecting plants to: (1) three mild (ID1) and severe (ID2) intermittent cycles of drought (7 days), each followed by rehydration (7 days); (2) mild (PD1) and severe (PD2) persistent drought; and (3) well-watered control (FI) during the vegetative growth stages (seedling, tillering, and jointing). The experiment was ended after 42 days of the implementation of the treatments. Changes in plant height (a), shoot dry Wt. (b), root dry Wt. (c), total dry mass (biomass) (d), and the root volume (e) are shown.

*Notes.* Bars represent means (±SD). Within each sampling (S) time, bars labelled with similar lowercase letter are not significantly different (p ≥ 0.05, LSD test). Asterisks denote significant differences: * (*p* < 0.05), ** (*p* < 0.01). Vertical dashed lines indicate the timing when pots subjected to ID1 and ID2 treatments entered the rehydration period (rehydration timing). Details of the FI, ID1, ID2, PD1, and PD2 treatments are provided in Figure 1.

**Figure S2.** Effects of drought treatments on interval-specific water use efficiency (WUEn) of balery plants (a) and the selected gas exchange parameter in barley leaves (b). Drought treatments were applied to 6-day-old seedlings, subjecting plants to: (1) three mild (ID1) and severe (ID2) intermittent cycles of drought (7 days) followed by rehydration (7 days); (2) persistent mild (PD1) and severe (PD2) drought; and (3) well-watered control (FI). The experiment was ended after 42 days of the implementation of the treatments. Interval-specific water use efficiency [WUEn (a)], and stomatal conductance [*g_s_* (b)] are shown.

*Notes.* Bars represent means (±SD). Within each sampling (S) time, bars labelled with similar lowercase letter are not significantly different (p ≥ 0.05, LSD test). Asterisks denote significant differences: * (*p* < 0.05), ** (*p* < 0.01). Vertical dashed lines indicate the timing when pots subjected to ID1 and ID2 treatments entered the rehydration period (rehydration timing). Details of the FI, ID1, ID2, PD1, and PD2 treatments are provided in Figure 1.

**Figure S3.** Composite image depicting the development of barley plants at various sampling times (S) under varied water availability regimes.

**
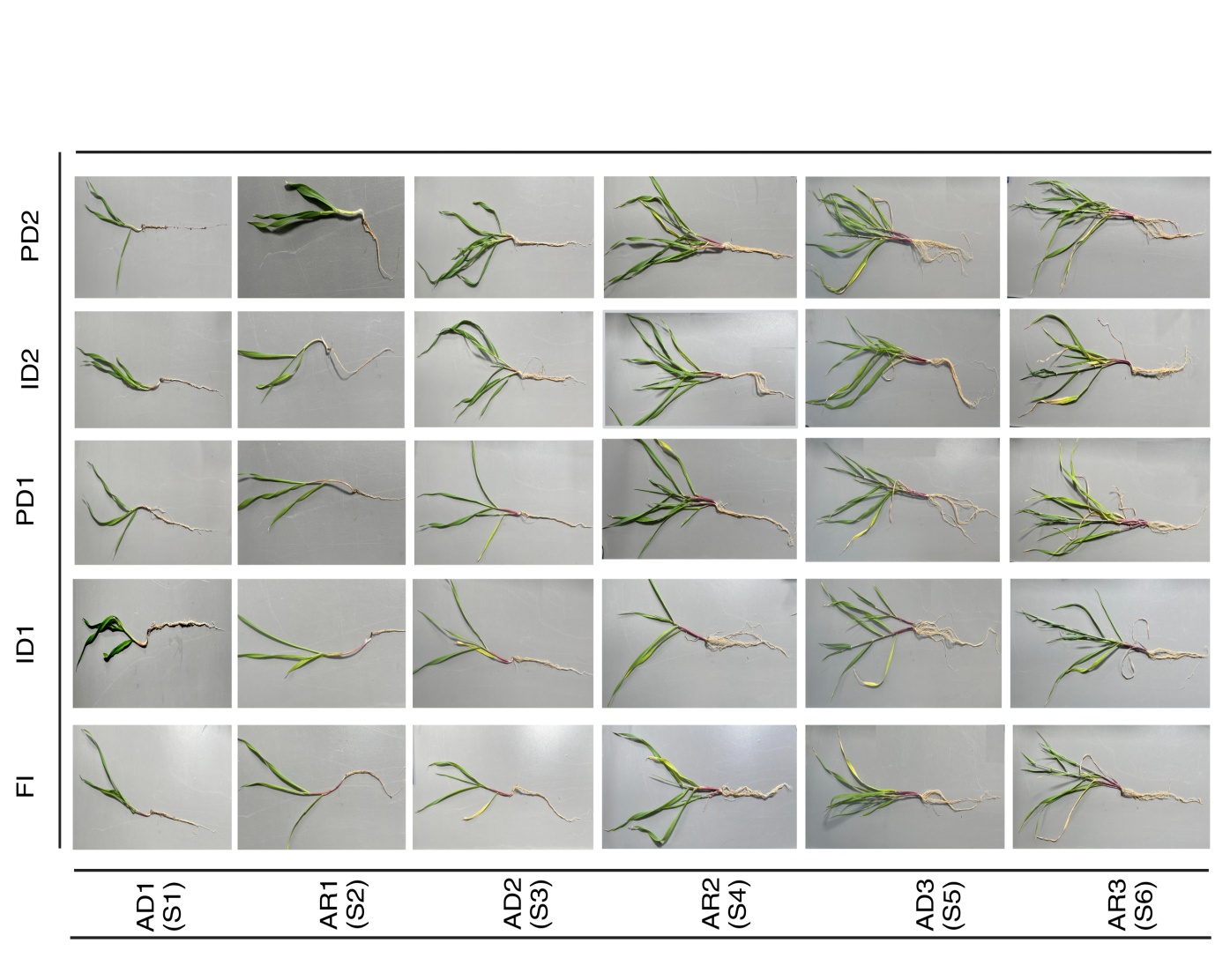
**

*Notes*. Details regarding drought stress treatments (FI, ID1, ID2, PD1, and PD2) and sampling times (AD1 (S1), AR1 (S2), AD2 (S3), AR2 (S4), AD3 (S5), and AR3 (S6)) are provided in Figure 1.

**Figure S4.** Shoot height of barley plants in response to varying types and levels of drought stress during the vegetative growth stages.


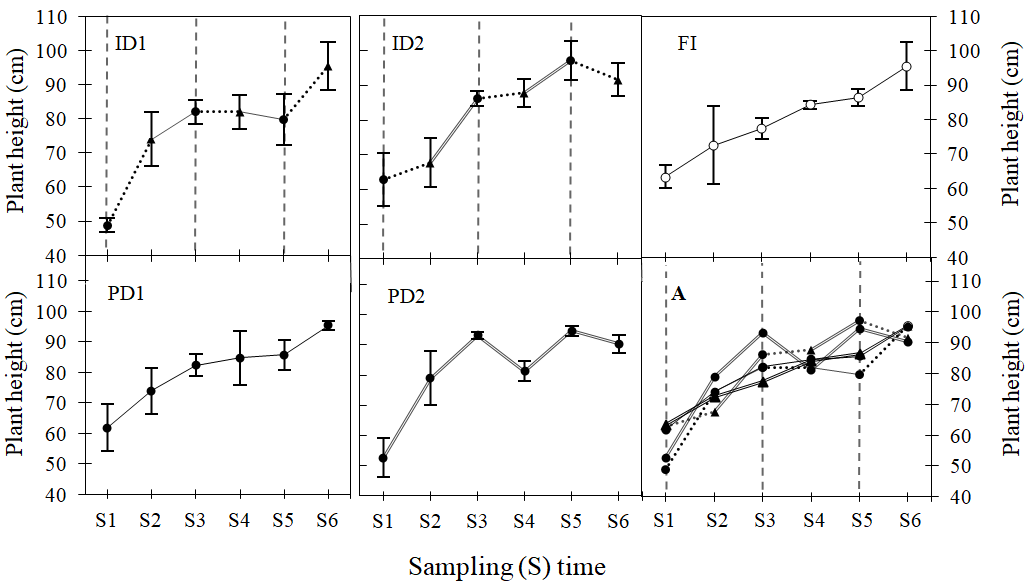


*Notes.* Details of the FI, ID1, ID2, PD1, and PD2 treatments and other information are provided in Figures 1 and 2. Markers represent mean values ± SD (n: 90 plants; 15 plants per sampling (S) time across 6 dates. Vertical dashed lines indicate the timing when pots subjected to ID1 and ID2 treatments entered the rehydration period (rehydration timing). Seedling stage (S1-S2), Tillering stage (S3-S4), Jointing stage (S5-S6). Image A (in Figure) shows all treatment trends combined in one graph.

**Figure S5.** Shoot/root ratio of barely plants in response to varying types and levels of drought stress during the vegetative growth stages.

**
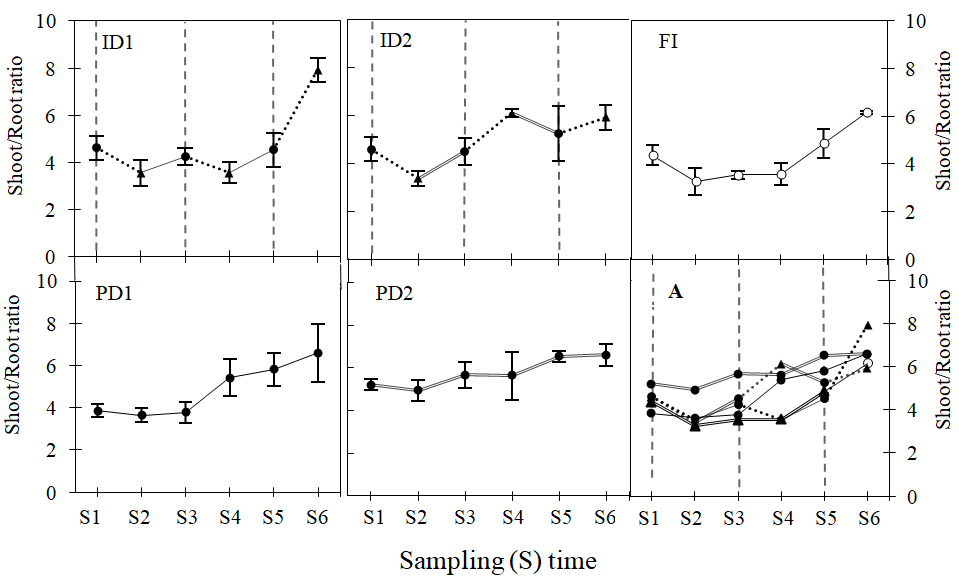
**

*Notes.* Details of the FI, ID1, ID2, PD1, and PD2 treatments and other information are provided in Figures 1 and 2. Markers represent mean values ± SD (n: 90 plants; 15 plants per sampling (S) time across 6 dates. Vertical dashed lines indicate the timing when pots subjected to ID1 and ID2 treatments entered the rehydration period (rehydration timing). Seedling stage (S1-S2), Tillering stage (S3-S4), Jointing stage (S5-S6). Image A (in Figure) shows all treatment trends combined in one graph.

**Figure S6.** Root volume of barley plants grown under varying types and levels of drought stress during the vegetative growth stages.


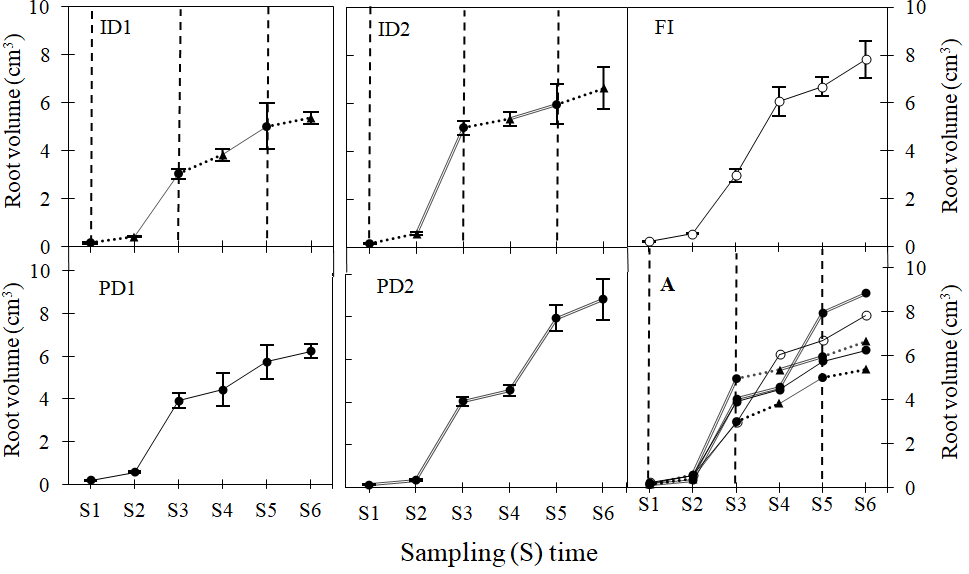


*Notes.* Details of the FI, ID1, ID2, PD1, and PD2 treatments and other information are provided in Figures 1 and 2. Markers represent mean values ± SD (n: 90 plants; 15 plants per sampling (S) time across 6 dates. Vertical dashed lines indicate the timing when pots subjected to ID1 and ID2 treatments entered the rehydration period (rehydration timing). Seedling stage (S1-S2), Tillering stage (S3-S4), Jointing stage (S5-S6). Image A (in Figure) shows all treatment trends combined in one graph.

**Figure S7** Tillering number (I) and schematic size (II) of barley plants in response to varying levels of drought stress during the vegetative growth stages.

*Notes.* Bars represent means (±SD). Within each sampling (S) time, bars labelled with similar lowercase letters are not significantly different (p ≥ 0.05, LSD test). Vertical dashed lines indicate the timing when pots subjected to ID1 and ID2 treatments entered the rehydration period (rehydration timing).  Absence of bars indicates no tiller production (zero tillering) at specific vegetative stages. Details of the FI, ID1, ID2, PD1, and PD2 treatments are provided in Figures 1 and 2.

**Figure S8** Total biomass, interval specific water-use efficiency (WUEn), and stomatal conductance (*gₛ*) in barely plants under FI and PD2 treatments.


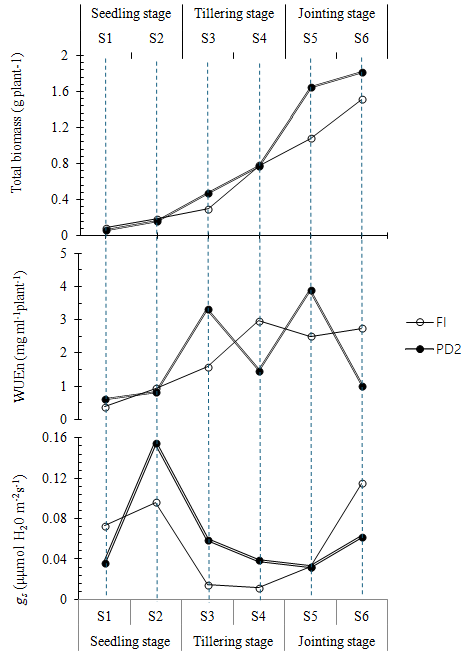


*Note***.** Details of the FI, and PD2 treatments are provided in Figure 1.

**Table.S1.** Mean squares from the ANOVA for plant height, root volume, root and shoot dry weight (Wt.), biomass, interval-specific water use efficiency (WUEn), and stomatal conductance (*g_s_*) in barley plants subjected to drought treatments (Data are given for different sampling times).

| Sampling (S) time | Source of variations | df |  | Plant height | Root  volume | Root dry Wt. | Shoot dry Wt. | Total biomass | WUEn | *g_s_* |
| --- | --- | --- | --- | --- | --- | --- | --- | --- | --- | --- |
| S1 | Drought | 4 |  | 134.192ns | 0.004** | 0.00003** | 0.00016ns | 0.00032* | 0.391** | 0.0023ns |
|  | Error | 10 |  | 34.783 | 0.0008 | 0.000002 | 0.00006 | 0.00008 | 0.00121 | 0.00069 |
|  | C.V (%) | - |  | 10.19 | 15.91 | 11.14 | 12.91 | 11.85 | 5.03 | 47.05 |
| S2 | Drought | 4 |  | 49.317ns | 0.0343** | 0.00018** | 0.0009ns | 0.00147ns | 0.1214* | 0.0025ns |
|  | Error | 10 |  | 75.167 | 0.0024 | 0.00002 | 0.00034 | 0.00043 | 0.0320 | 0.00093 |
|  | C.V (%) | - |  | 11.81 | 10.12 | 10.05 | 12.56 | 11.53 | 21.17 | 22.58 |
| S3 | Drought | 4 |  | 103.108** | 2.051** | 0.0003* | 0.015** | 0.018** | 22.546** | 0.0011** |
|  | Error | 10 |  | 8.150 | 0.072 | 0.00009 | 0.0016 | 0.0022 | 1.342 | 0.00009 |
|  | C.V (%) | - |  | 3.39 | 7.10 | 13.31 | 12.66 | 12.01 | 30.04 | 30.15 |
| S4 | Drought | 4 |  | 19.767ns | 2.292** | 0.0021ns | 0.031ns | 0.034ns | 1.349** | 0.0003** |
|  | Error | 10 |  | 26.717 | 0.240 | 0.0009 | 0.009 | 0.0142 | 0.2133 | 0.00002 |
|  | C.V (%) | - |  | 6.15 | 10.10 | 23.54 | 15.98 | 16.56 | 23.41 | 22.25 |
| S5 | Drought | 4 |  | 152.767** | 3.665** | 0.0034ns | 0.295** | 0.345** | 1.333ns | 0.00021* |
|  | Error | 10 |  | 24.083 | 0.558 | 0.001 | 0.026 | 0.312 | 0.478 | 0.0001 |
|  | C.V (%) | - |  | 5.53 | 11.92 | 18.39 | 17.07 | 15.72 | 24.97 | 26.47 |
| S6 | Drought | 4 |  | 18.458ns | 5.507** | 0.0026** | 0.157** | 0.177** | 7.574** | 0.0028** |
|  | Error | 10 |  | 26.100 | 0.486 | 0.0004 | 0.023 | 0.022 | 0.658 | 0.0001 |
|  | C.V (%) | - |  | 5.45 | 9.99 | 10.96 | 11.67 | 10.00 | 38.94 | 15.66 |

*Note.* * – p < 0.05, ** – p < 0.01, ns – p > 0.05; df, degrees of freedom; C.V., coefficient of variation.
